# Nomenclature for Factors of the HLA System, Update October, November and December 2024

**DOI:** 10.1111/iji.12707

**Published:** 2025-01-28

**Authors:** Steven G. E. Marsh

**Affiliations:** ^1^ UCL Cancer Institute, University College London (UCL) London UK

1

The following sequences have been submitted to the Nomenclature Committee since the July, August and September 2024 nomenclature update (Marsh, 2024) and, following agreed policy, have been assigned official allele designations (Marsh et al. 2010). Full details of all sequences will be published in a forthcoming report.

Below are listed the newly assigned sequences (Table 1) and confirmations of previously reported sequences (Table 2). The accession number of each sequence is given, and these can be used to retrieve the sequence files from the EMBL, GenBank or DDBJ data libraries. Although accession numbers have been assigned by the data libraries and most sequences are already available, there is still the possibility that an author may not yet have allowed the sequence to be released; in such a case, you will have to contact the submitting author directly. Additional information pertaining to new sequences is often included in the publications describing these alleles; a listing of recent publications that describe new HLA sequences is given in Table 3.

**TABLE 1:** New sequences.

HLA allele	Cell identification	Accession number	Submitting author
A*01:471	FG8734	PQ325569	Dr Thibault Pajot, Lille, France
A*01:472	HG00052660	PQ310439	Histogenetics, Ossining, NY, USA
A*01:473Q	022024514	PQ286036	Mrs Ornella Senoussi, La Tronche, France
A*01:474	L_109	PQ300650	Dr Samir Agarwal, New Delhi, India
A*01:475	KSR206847	PQ479083	Dr Maria Loginova, Kirov, Russia
A*01:476Q	KSR187914	PQ479084	Dr Maria Loginova, Kirov, Russia
A*01:477	HG00055219	PQ497700	Histogenetics, Ossining, NY, USA
A*01:478	24‐1472	PQ568070	Dr M. Rosa Moya‐Quiles, El Palmar, Spain
A*02:01:01:257	204‐01535	PQ212803	Miss Yue Han, Xi'an, China
A*02:05:15	RP‐285161	PQ285422	Dr Neifi Hassan Saloum Deghaide, Ribeirao Preto, Brazil
A*02:06:01:17	2023111424	MZ714558	Dr Mingrui Huo, Beijing, China
A*02:07:01:07	LJG‐01	PP712097	Dr Tie Cheng Sun, Beijing, China
A*02:1026:02	E000624062696	PQ603088	Dr Dolores Planelles, Valencia, Spain
A*02:1195N	HB18240202	PP574991	Dr Justine Schmitt, Liege, Belgium
A*02:1196	UERJ‐C20597‐A	PQ162437	Dr Romulo Vianna, Rio de Janeiro, Brazil
A*02:1197	RP‐282241	PQ274246	Dr Neifi Hassan Saloum Deghaide, Ribeirao Preto, Brazil
A*02:1198	H8108	PQ323357	Dr Vincent Elsermans, Lille cedex, France
A*02:1198	G4150	PQ325570	Dr Thibault Pajot, Lille, France
A*02:1199	65231179229	PQ381334	Mr Gerald Bertrand, Rennes Cedex, France
A*02:1200	rdkm3558100	PP982200	Ms Anastasiia Ananeva, Moscow, Russia
A*02:1201	HG00055218	PQ497699	Histogenetics, Ossining, NY, USA
A*02:1202	HG00055215	PQ481495	Histogenetics, Ossining, NY, USA
A*02:1203	2022101387	MZ714558	Dr Mingrui Huo, Beijing, China
A*02:1204	1811/24	PQ541131	Mr Rafael Oliveira, Vitoria, Brazil
A*02:1205	900612490071266	PQ535693	Prof Faming Zhu, Hangzhou, China
A*02:1206	mvz‐mrr_656987	OZ199514	Miss Kristin Kipper, Martinsried, Germany
A*03:01:01:123	C00062314120242	PQ241319	Dr William Lemieux, Saint‐Laurent, Canada
A*03:498	021754896	PQ280737	Dr Margot Lepage, Decines‐Charpieu, France
A*03:499	1123052521	PQ381333	Mr Gerald Bertrand, Rennes Cedex, France
A*03:500	HG00055217	PQ497698	Histogenetics, Ossining, NY, USA
A*03:501Q	HG00055216	PQ481496	Histogenetics, Ossining, NY, USA
A*03:502	GLAS99623	PQ565819	Dr Claire Walker, Glasgow, UK
A*03:503	31200200312	PP105531	Dr Jigarkumar Suthar, Ahmedabad, India
A*03:504N	HG00055256	PQ619031	Histogenetics, Ossining, NY, USA
A*11:01:01:93	202403803	PQ305752	Dr Amalia Tejeda Velarde, Salamanca, Spain
A*11:481	2023022064	MZ714556	Dr Mingrui Huo, Beijing, China
A*11:482	KSR137199	PQ479085	Dr Maria Loginova, Kirov, Russia
A*11:483	HG00055241	PQ535515	Histogenetics, Ossining, NY, USA
A*11:484	A‐5963010101321260	PP781550	Dr Elena Khromova, Saint Petersburg, Russian Federation
A*11:485	WC‐4189	PQ628235	Dr Tie Cheng Sun, Beijing, China
A*11:486	HG00055257	PQ655354	Histogenetics, Ossining, NY, USA
A*23:148	2488319	PQ492166	Mrs Ana Lucia Rodrigues, Aparecida de Goiania, Brazil
A*23:149	HG00055240	PQ535514	Histogenetics, Ossining, NY, USA
A*24:02:01:145	NGS‐1094d1	PQ336938	Dr Meenaskshi Singh, Navi Mumbai, India
A*24:02:177	20240219047	PQ212876	Dr Mingrui Huo, Beijing, China
A*24:02:178	rdkm168162	PP982201	Ms Anastasiia Ananeva, Moscow, Russia
A*24:07:05	NGS‐1082D2	PQ336937	Dr Meenaskshi Singh, Navi Mumbai, India
A*24:452:02	HG00052661	PQ310440	Histogenetics, Ossining, NY, USA
A*24:643	NGS‐1195D5	PQ336941	Dr Meenaskshi Singh, Navi Mumbai, India
A*24:644	HG00052659	PQ310438	Histogenetics, Ossining, NY, USA
A*24:645	HG00055204	PQ369420	Histogenetics, Ossining, NY, USA
A*24:646	rdkm302161	PP974457	Ms Anastasiia Ananeva, Moscow, Russia
A*24:647	A‐292810101077179561	PQ050372	Dr Elena Khromova, Saint Petersburg, Russian Federation
A*24:648	HG00055254	PQ594986	Histogenetics, Ossining, NY, USA
A*25:01:22	HG00055203	PQ369419	Histogenetics, Ossining, NY, USA
A*25:93	KSR206412	PQ479086	Dr Maria Loginova, Kirov, Russia
A*25:94	119249	PQ505996	Dr Francisco de Paula Sanchez Gordo, Malaga, Spain
A*26:01:86	HG00055258	PQ655355	Histogenetics, Ossining, NY, USA
A*26:250:01:02	65220403520	PQ381325	Mr Gerald Bertrand, Rennes Cedex, France
A*26:253	017142229	PQ274447	Dr Margot Lepage, Decines‐Charpieu, France
A*29:199N	2461719	PQ282132	Mrs Ana Lucia Rodrigues, Aparecida de Goiania, Brazil
A*29:200N	FA5723	PQ323368	Dr Thibault Pajot, Lille, France
A*30:01:31	81351415	PQ468470	Dr Daniel Fuerst, Ulm, Germany
A*30:02:33	1819341	PP975066	Ms Gisele Rampim, Sao Paulo, Brazil
A*30:233	NT02528	OR497365	Dr Jennifer Ng, Rockville, MD, USA
A*30:234	LIB_6172832	PP996047	Ms Anaregina Araujo, Teresina, Brazil
A*30:235	INCA1075210	PQ283680	Mr Vinicius Stelet, Rio de Janeiro, Brazil
A*30:236	2487566	PQ461652	Mrs Ana Lucia Rodrigues, Aparecida de Goiania, Brazil
A*30:237	2331559	PQ492168	Mrs Ana Lucia Rodrigues, Aparecida de Goiania, Brazil
A*31:235	Tsimnovel8	PP855437	Assoc Prof Paul Norman, Aurora, USA
A*32:01:62	R8841	PQ346950	Dr Thibault Pajot, Lille, France
A*32:01:63	24222114	PQ435225	Dr Daniel Fuerst, Ulm, Germany
A*32:191Q	1122119871	PQ381329	Mr Gerald Bertrand, Rennes Cedex, France
A*33:03:01:28	NGS‐1042D3	PQ336936	Dr Meenaskshi Singh, Navi Mumbai, India
A*33:275	HG00055211	PQ414909	Histogenetics, Ossining, NY, USA
A*33:276	HG00055255	PQ594987	Histogenetics, Ossining, NY, USA
A*68:01:68	HG00055214	PQ414912	Histogenetics, Ossining, NY, USA
A*68:01:69	scu04891tu	PQ633300	Dr Francisco de Paula Sanchez Gordo, Malaga, Spain
A*68:02:26	HSL_002	PQ329215	Dr Melchior Le Mene, Paris, France
A*68:91:02	KSR167983	PQ479087	Dr Maria Loginova, Kirov, Russia
A*68:324	022088059	PQ280739	Dr Margot Lepage, Decines‐Charpieu, France
A*68:325	J8697	PQ286539	Dr Thibault Pajot, Lille, France
A*68:326	HSL_003	PQ329216	Dr Melchior Le Mene, Paris, France
A*69:01:06	42433075	PQ310667	Dr Romain Ferru‐Clement, Poitiers, France
A*74:52	06227905	PQ215699	Mr Abootaleb Rahmanian, Portland, USA
B*07:02:01:130Q	24224471	PQ583038	Dr Daniel Fuerst, Ulm, Germany
B*07:02:108	DKMS‐LSL_ID20052549	OZ174404	DKMS Life Sciences Lab., Dresden, Germany
B*07:516:01:02	65230341079	PQ381331	Mr Gerald Bertrand, Rennes Cedex, France
B*07:518	DKMS‐LSL_ID20281978	OZ174442	DKMS Life Sciences Lab., Dresden, Germany
B*07:519	DKMS‐LSL_ID21197556	OZ174425	DKMS Life Sciences Lab., Dresden, Germany
B*07:520N	DKMS‐LSL_ID20026171	OZ174400	DKMS Life Sciences Lab., Dresden, Germany
B*07:521	021244545	PQ280736	Dr Margot Lepage, Decines‐Charpieu, France
B*07:522	41821143	PQ310669	Dr Romain Ferru‐Clement, Poitiers, France
B*07:523	022107151	PQ286039	Mrs Ornella Senoussi, La Tronche, France
B*07:524	1451/24	PQ541130	Mr Rafael Oliveira, Vitoria, Brazil
B*07:525	KSR104739	PQ511336	Dr Maria Loginova, Kirov, Russia
B*08:01:01:76	DKMS‐LSL_ID19845219	OZ174390	DKMS Life Sciences Lab., Dresden, Germany
B*08:01:79	HG00052662	PQ310453	Histogenetics, Ossining, NY, USA
B*08:111:02	DKMS‐LSL_ID20034872	OZ174402	DKMS Life Sciences Lab., Dresden, Germany
B*08:281:02	GLAS96284	PQ565821	Dr Claire Walker, Glasgow, UK
B*08:327	DKMS‐LSL_ID20055656	OZ174405	DKMS Life Sciences Lab., Dresden, Germany
B*08:328N	DKMS‐LSL_ID20289922	OZ177833	DKMS Life Sciences Lab., Dresden, Germany
B*08:329Q	DKMS‐LSL_ID20010142	OZ177829	DKMS Life Sciences Lab., Dresden, Germany
B*08:330	HG00055205	PQ369421	Histogenetics, Ossining, NY, USA
B*08:331	2205736	PQ317781	Dr Mirzokhid Rakhmanov, Freiburg, Germany
B*08:332	KSR183302	PQ511337	Dr Maria Loginova, Kirov, Russia
B*13:02:01:31	5345634	PP928410	Prof Renata Zunec, Zagreb, Croatia
B*13:203	65230746084	PQ381332	Mr Gerald Bertrand, Rennes Cedex, France
B*14:02:32	DKMS‐LSL_ID20102660	OZ174380	DKMS Life Sciences Lab., Dresden, Germany
B*14:02:33	rdkm509131	PP974449	Ms Anastasiia Ananeva, Moscow, Russia
B*14:135	DKMS‐LSL_ID20762728	OZ174434	DKMS Life Sciences Lab., Dresden, Germany
B*14:136	2024‐0612	PQ300632	Dr Qingdong Guan, Winnipeg, Canada
B*14:137	KSR113968	PQ511338	Dr Maria Loginova, Kirov, Russia
B*14:138	HG00055271	PQ655362	Histogenetics, Ossining, NY, USA
B*14:139N	HG00055260	PQ594957	Histogenetics, Ossining, NY, USA
B*15:01:01:70	DKMS‐LSL_ID19958491	OZ174394	DKMS Life Sciences Lab., Dresden, Germany
B*15:01:90	G231221010	OZ060361	Miss Xingjie Li, Chengdu, China
B*15:01:91	Tsimnovel7	PP855435	Assoc Prof Paul Norman, Aurora, USA
B*15:78:05	42318071	PQ563458	Dr Romain Ferru‐Clement, Poitiers, France
B*15:628:02	HG00055263, HG00055264	PQ619032, PQ619033	Histogenetics, Ossining, NY, USA
B*15:720	NTV_6100363	PQ195599	Dr Georgia Daniela Marcusso, Porto Velho, Brazil
B*15:721	DKMS‐LSL_ID20128312	OZ174410	DKMS Life Sciences Lab., Dresden, Germany
B*15:722	INCA1075666	PQ510362	Mr Vinicius Stelet, Rio de Janeiro, Brazil
B*15:723	KSR170321	PQ511339	Dr Maria Loginova, Kirov, Russia
B*15:724	HG00055259	PQ594956	Histogenetics, Ossining, NY, USA
B*18:01:01:91	DKMS‐LSL_ID21596017	OZ174417	DKMS Life Sciences Lab., Dresden, Germany
B*18:01:55	5334802	PQ112524	Prof Renata Zunec, Zagreb, Croatia
B*18:01:56	RMU4869	PQ360871	Mr Valery Cheranev, Moscow, Russia
B*18:01:57	rdkm454160	PP974452	Ms Anastasiia Ananeva, Moscow, Russia
B*18:01:58	24219918	PQ310781	Dr Daniel Fuerst, Ulm, Germany
B*18:01:59	KSR114614	PQ511340	Dr Maria Loginova, Kirov, Russia
B*18:251	DKMS‐LSL_ID19860133	OZ177826	DKMS Life Sciences Lab., Dresden, Germany
B*18:252	202412457	PQ305751	Dr Amalia Tejeda Velarde, Salamanca, Spain
B*18:253	HG00055206	PQ369422	Histogenetics, Ossining, NY, USA
B*18:254	41926052	PQ588447	Dr Romain Ferru‐Clement, Poitiers, France
B*27:02:08	DKMS‐LSL_ID21157558	OZ174385	DKMS Life Sciences Lab., Dresden, Germany
B*27:05:02:44	BMT_3107_SARR	PP471571	Dr Samir Agarwal, New Delhi, India
B*27:05:02:45	DKMS‐LSL_ID20119781	OZ174382	DKMS Life Sciences Lab., Dresden, Germany
B*27:284	B0702020011	PP481374	Dr Justine Schmitt, Liege, Belgium
B*27:285	DKMS‐LSL_ID21287989	OZ174419	DKMS Life Sciences Lab., Dresden, Germany
B*27:286	65231368169	PQ381338	Mr Gerald Bertrand, Rennes Cedex, France
B*27:287N	HG00055268	PQ619037	Histogenetics, Ossining, NY, USA
B*35:01:01:90	71616508	PQ218691	Dr Luis A. Marin, Albacete, Spain
B*35:01:01:91	GR‐EVA‐59967	PQ498406	Dr Diamanto Kouniaki, Athens, Greece
B*35:01:79	DKMS‐LSL_ID20182147	OZ174413	DKMS Life Sciences Lab., Dresden, Germany
B*35:01:80	RMU8072	PQ360873	Mr Valery Cheranev, Moscow, Russia
B*35:01:81	rdkm586155	PP974453	Ms Anastasiia Ananeva, Moscow, Russia
B*35:01:82	KSR220655	PQ511341	Dr Maria Loginova, Kirov, Russia
B*35:02:24	HG00055269	PQ619038	Histogenetics, Ossining, NY, USA
B*35:05:07	502426	PQ533674	Mr Alessandro Pirri, Curitiba, Brazil
B*35:31:01:02	24404620	PQ479081	Dr Luis A. Marin, Albacete, Spain
B*35:622	DKMS‐LSL_ID20110905	OZ174408	DKMS Life Sciences Lab., Dresden, Germany
B*35:623	DKMS‐LSL_ID21199936	OZ174424	DKMS Life Sciences Lab., Dresden, Germany
B*35:624	DKMS‐LSL_ID20032851	OZ174401	DKMS Life Sciences Lab., Dresden, Germany
B*35:625	DKMS‐LSL_ID19801712	OZ174388	DKMS Life Sciences Lab., Dresden, Germany
B*35:626	DKMS‐LSL_ID20288120	OZ177832	DKMS Life Sciences Lab., Dresden, Germany
B*35:627	Y8395	PQ286540	Dr Thibault Pajot, Lille, France
B*35:628	FG8281	PQ325571	Dr Thibault Pajot, Lille, France
B*35:629N	2023032172	MZ714558	Dr Mingrui Huo, Beijing, China
B*35:630	65221301665	PQ381330	Mr Gerald Bertrand, Rennes Cedex, France
B*35:631	rdkm219147	PP974450	Ms Anastasiia Ananeva, Moscow, Russia
B*35:632	rdkm989147	PP995159	Ms Anastasiia Ananeva, Moscow, Russia
B*35:633	NCY_B_PDS_01	PQ248966	Dr Adele Dhuyser, Vandoeuvre Les Nancy, France
B*35:634	HG00055243	PQ535511	Histogenetics, Ossining, NY, USA
B*35:635	Tsimnovel9	PP855436	Assoc Prof Paul Norman, Aurora, USA
B*35:636N	KSR130208, KSR179333	PQ511342	Dr Maria Loginova, Kirov, Russia
B*37:01:33	DKMS‐LSL_ID19988671	OZ177828	DKMS Life Sciences Lab., Dresden, Germany
B*37:01:34	DKMS‐LSL_ID20252415	OZ177831	DKMS Life Sciences Lab., Dresden, Germany
B*37:114	23B71640	PP911332	Dr Yajie You, Nanjing City, China
B*37:115	DKMS‐LSL_ID20470913	OZ174438	DKMS Life Sciences Lab., Dresden, Germany
B*37:116	DKMS‐LSL_ID21278146	OZ174420	DKMS Life Sciences Lab., Dresden, Germany
B*37:117	1122055181	PQ381326	Mr Gerald Bertrand, Rennes Cedex, France
B*37:118	GLAS95185	PQ565820	Dr Claire Walker, Glasgow, UK
B*37:119	BMT_3447	PQ634767	Dr Samir Agarwal, New Delhi, India
B*38:01:01:20	5345510	PP928415	Prof Renata Zunec, Zagreb, Croatia
B*38:01:26	10101012395455	PQ360869	Mr Valery Cheranev, Moscow, Russia
B*38:02:15	DKMS‐LSL_ID20118155	OZ174409	DKMS Life Sciences Lab., Dresden, Germany
B*38:120	DKMS‐LSL_ID21478174	OZ174386	DKMS Life Sciences Lab., Dresden, Germany
B*38:121	M8591	PQ323364	Dr Thibault Pajot, Lille, France
B*38:122	GUFM‐001	PQ394279	Dr Sevim Gonen, Ankara, Turkey
B*39:01:42	DKMS‐LSL_ID20838124	OZ174444	DKMS Life Sciences Lab., Dresden, Germany
B*39:13:03	RP‐284990	PQ274245	Dr Neifi Hassan Saloum Deghaide, Ribeirao Preto, Brazil
B*39:227	KSR128348	PQ511343	Dr Maria Loginova, Kirov, Russia
B*39:228	119621	PQ633301	Dr Francisco de Paula Sanchez Gordo, Malaga, Spain
B*40:01:91	rdkm605136	PQ014270	Ms Anastasiia Ananeva, Moscow, Russia
B*40:01:92	KSRLarkovDS, KSRLarkovSN	PQ511344	Dr Maria Loginova, Kirov, Russia
B*40:02:41	rdkm424166	PQ014269	Ms Anastasiia Ananeva, Moscow, Russia
B*40:06:01:20	24817626	PQ383847	Dr Luis A. Marin, Albacete, Spain
B*40:597	DKMS‐LSL_ID19991408	OZ174379	DKMS Life Sciences Lab., Dresden, Germany
B*40:598	DKMS‐LSL_ID20286262	OZ174383	DKMS Life Sciences Lab., Dresden, Germany
B*40:599	DKMS‐LSL_ID19819234	OZ174389	DKMS Life Sciences Lab., Dresden, Germany
B*40:600Q	LIB_5989973	PQ457082	Ms Anaregina Araujo, Teresina, Brazil
B*40:601	2023050965	PQ247469	Dr Mingrui Huo, Beijing, China
B*40:602	KSR195824	PQ511345	Dr Maria Loginova, Kirov, Russia
B*41:01:01:09	24404833	PQ634796	Dr Luis A. Marin, Albacete, Spain
B*41:02:13	rdkm948126	PQ014271	Ms Anastasiia Ananeva, Moscow, Russia
B*41:88	HG00055221	PQ481498	Histogenetics, Ossining, NY, USA
B*42:39	rdkm532146	PQ014268	Ms Anastasiia Ananeva, Moscow, Russia
B*44:02:89	DKMS‐LSL_ID20216789	OZ174414	DKMS Life Sciences Lab., Dresden, Germany
B*44:02:90	510185	PQ351809	Mr Alessandro Pirri, Curitiba, Brazil
B*44:414	rdkm641169	PP501823	Ms Anastasiia Ananeva, Moscow, Russia
B*44:415	DKMS‐LSL_ID20136413	OZ174411	DKMS Life Sciences Lab., Dresden, Germany
B*44:416	DKMS‐LSL_ID19885865	OZ174391	DKMS Life Sciences Lab., Dresden, Germany
B*44:417	G4395	PQ286541	Dr Thibault Pajot, Lille, France
B*44:418	101261	PQ283401	Mr Valery Cheranev, Moscow, Russia
B*44:419	65220403061	PQ381324	Mr Gerald Bertrand, Rennes Cedex, France
B*44:420	rdkm085166	PQ014272	Ms Anastasiia Ananeva, Moscow, Russia
B*44:421	KSR163566	PQ511346	Dr Maria Loginova, Kirov, Russia
B*44:422	KSR133106	PQ511346	Dr Maria Loginova, Kirov, Russia
B*44:423	HG00055270	PQ655361	Histogenetics, Ossining, NY, USA
B*44:424	HG00055266	PQ619035	Histogenetics, Ossining, NY, USA
B*46:103	HG00052664	PQ346913	Histogenetics, Ossining, NY, USA
B*48:65	rdkm760154	PP501821	Ms Anastasiia Ananeva, Moscow, Russia
B*48:66	510850	PQ586425	Mr Alessandro Pirri, Curitiba, Brazil
B*48:67	HG00055265	PQ619034	Histogenetics, Ossining, NY, USA
B*49:01:01:23	0123260007345	OR725007	Mrs Hanan Al Anazi, Riyadh, Saudi Arabia
B*49:88	DKMS‐LSL_ID20111347	OZ177830	DKMS Life Sciences Lab., Dresden, Germany
B*50:28	LIB_5943313	PQ473724	Ms Anaregina Araujo, Teresina, Brazil
B*51:01:01:124	NGS‐1179D1	PQ336940	Dr Meenaskshi Singh, Navi Mumbai, India
B*51:01:01:125	2304914	PQ317782	Dr Mirzokhid Rakhmanov, Freiburg, Germany
B*51:01:112	HG00055220	PQ481497	Histogenetics, Ossining, NY, USA
B*51:417	DKMS‐LSL_ID20230856	OZ174443	DKMS Life Sciences Lab., Dresden, Germany
B*51:418	DKMS‐LSL_ID20009049	OZ174397	DKMS Life Sciences Lab., Dresden, Germany
B*51:419	65230692493	PQ381337	Mr Gerald Bertrand, Rennes Cedex, France
B*51:420	rdkm452160	PP974451	Ms Anastasiia Ananeva, Moscow, Russia
B*51:421	KSR219308	PQ511350	Dr Maria Loginova, Kirov, Russia
B*51:422	HG00055261	PQ594958	Histogenetics, Ossining, NY, USA
B*52:128	DKMS‐LSL_ID19785769	OZ174387	DKMS Life Sciences Lab., Dresden, Germany
B*52:129	DKMS‐LSL_ID20052420	OZ174403	DKMS Life Sciences Lab., Dresden, Germany
B*52:130	RMU773	PQ360870	Mr Valery Cheranev, Moscow, Russia
B*53:75:01:02	DKMS‐LSL_ID19985413	OZ177827	DKMS Life Sciences Lab., Dresden, Germany
B*55:01:32	DKMS‐LSL_ID19911169	OZ174392	DKMS Life Sciences Lab., Dresden, Germany
B*55:01:33	DKMS‐LSL_ID21180004	OZ174427	DKMS Life Sciences Lab., Dresden, Germany
B*55:142	DKMS‐LSL_ID21170218	OZ174429	DKMS Life Sciences Lab., Dresden, Germany
B*56:102	017875986	PQ476022	Dr Xavier Fournel, Saint Etienne, France
B*57:186	DKMS‐LSL_ID21170858	OZ174428	DKMS Life Sciences Lab., Dresden, Germany
B*57:187	DKMS‐LSL_ID19902987	OZ174378	DKMS Life Sciences Lab., Dresden, Germany
B*57:188	DKMS‐LSL_ID20059927	OZ174407	DKMS Life Sciences Lab., Dresden, Germany
B*57:189	DKMS‐LSL_ID21208194	OZ174423	DKMS Life Sciences Lab., Dresden, Germany
B*57:190	017837901	PQ280738	Dr Margot Lepage, Decines‐Charpieu, France
B*57:190	DKMS‐LSL_ID20774919	OZ177835	DKMS Life Sciences Lab., Dresden, Germany
B*57:191	HG00055244	PQ535512	Histogenetics, Ossining, NY, USA
B*58:01:01:21	DKMS‐LSL_ID20499295	OZ174440	DKMS Life Sciences Lab., Dresden, Germany
B*58:01:50	464882	OR204704	Prof Edward KL Yang, Hualien, Taiwan
B*58:01:51	317013	PQ357059	Dr Raquel Fabreti, Belo Horizonte, Brazil
B*58:157	DKMS‐LSL_ID19922353	OZ174393	DKMS Life Sciences Lab., Dresden, Germany
B*58:158	HG00052663	PQ346912	Histogenetics, Ossining, NY, USA
B*58:159	2024‐0753	PQ603089	Dr Qingdong Guan, Winnipeg, Canada
B*81:16	DKMS‐LSL_ID20059119	OZ174406	DKMS Life Sciences Lab., Dresden, Germany
C*01:02:01:78	GR‐EVA‐58952	PQ261106	Dr Diamanto Kouniaki, Athens, Greece
C*01:02:01:79Q	rdkm197154	PQ041715	Ms Anastasiia Ananeva, Moscow, Russia
C*01:02:95	rdkm196100	PP947783	Ms Anastasiia Ananeva, Moscow, Russia
C*01:280	KSR175796	PQ240367	Dr Maria Loginova, Kirov, Russia
C*01:281	KUMC‐E‐003	PQ261032	Mrs So‐Jung Choi, Seoul, South Korea
C*01:282	21‐1011	PQ550136	Assoc Prof Ahmed Mostafa, Saskatoon, Canada
C*01:283	6208598‐BLS	PQ159733	Dr Jeane Visentainer, Maringa, Brazil
C*01:284	9000624505267092	PQ535695	Prof Faming Zhu, Hangzhou, China
C*02:10:01:20	100424404965	PQ463668	Dr Luis A. Marin, Albacete, Spain
C*02:37:02	HG00055225	PQ497696	Histogenetics, Ossining, NY, USA
C*02:251	65220160522	PQ268864	Mr Gerald Bertrand, Rennes Cedex, France
C*02:252	rdkm702164	PP947787	Ms Anastasiia Ananeva, Moscow, Russia
C*02:253N	A‐5094778022400151401	PQ438943	Dr Elena Khromova, Saint Petersburg, Russian Federation
C*03:02:35	24219187	PQ360896	Dr Daniel Fuerst, Ulm, Germany
C*03:04:02:06	24221857	PQ435224	Dr Daniel Fuerst, Ulm, Germany
C*03:536:02	900612190062326	PQ535696	Prof Faming Zhu, Hangzhou, China
C*03:698	KSR142917	PQ240369	Dr Maria Loginova, Kirov, Russia
C*03:699	KSR91973, KSR168829	PQ240370	Dr Maria Loginova, Kirov, Russia
C*03:700	KSR157847	PQ240371	Dr Maria Loginova, Kirov, Russia
C*03:701	65220255433	PQ268863	Mr Gerald Bertrand, Rennes Cedex, France
C*03:702	RMU7547	PQ360872	Mr Valery Cheranev, Moscow, Russia
C*03:703	HD8018	PQ110589	Dr Vincent Elsermans, Lille cedex, France
C*04:01:01:194Q	1171076	PQ304401	Dr Antonio Balas, Madrid, Spain
C*04:01:168	HG00052667	PQ310442	Histogenetics, Ossining, NY, USA
C*04:533	240701234	PQ035929	Dr Francesco Ingrassia, Palermo, Italy
C*04:534	2402210	PQ346315	Dr Paul Rouzaire, Clermont‐Ferrand, France
C*04:535	S65	LC846669	Prof Takashi Shiina, Isehara, Japan
C*04:536	2489813	PQ461651	Mrs Ana Lucia Rodrigues, Aparecida de Goiania, Brazil
C*04:537	2491926	PQ492169	Mrs Ana Lucia Rodrigues, Aparecida de Goiania, Brazil
C*04:538	D00980174	PQ619696	Dr Michele Curcio, Pisa, Italy
C*04:539	HG00055274	PQ594962	Histogenetics, Ossining, NY, USA
C*05:01:83	G4398	PQ323366	Dr Thibault Pajot, Lille, France
C*05:01:84	NRCH00044	PP982820	Mr Evgeny Leonov, Moscow, Russia
C*05:303N	KSR89052	PQ240373	Dr Maria Loginova, Kirov, Russia
C*05:304	HG00055247	PQ535524	Histogenetics, Ossining, NY, USA
C*05:305	RP‐287131	PQ568229	Dr Neifi Hassan Saloum Deghaide, Ribeirao Preto, Brazil
C*05:306	HG00055278	PQ655357	Histogenetics, Ossining, NY, USA
C*06:02:01:102	NGS‐954D1, NGS‐954	PQ336935	Dr Meenaskshi Singh, Navi Mumbai, India
C*06:02:01:103	NGS‐1147	PQ336939	Dr Meenaskshi Singh, Navi Mumbai, India
C*06:02:119	KSR197787	PQ329220	Dr Maria Loginova, Kirov, Russia
C*06:02:120	HG00055224	PQ481499	Histogenetics, Ossining, NY, USA
C*06:395	900612490025526	PQ118951	Prof Faming Zhu, Hangzhou, China
C*06:396Q	KSR111771	PQ329218	Dr Maria Loginova, Kirov, Russia
C*06:397	KSR107046	PQ329219	Dr Maria Loginova, Kirov, Russia
C*06:398	rdkm318110	PP947785	Ms Anastasiia Ananeva, Moscow, Russia
C*06:399	HG00055207	PQ369423	Histogenetics, Ossining, NY, USA
C*07:02:01:205	5346320	PP928411	Prof Renata Zunec, Zagreb, Croatia
C*07:02:161	KSR138956	PQ329224	Dr Maria Loginova, Kirov, Russia
C*07:02:162	FND00005362	PP763745	Dr Eugene Albert, Dolgoprudny, Russian Federation
C*07:1164	2464806	PQ282130	Mrs Ana Lucia Rodrigues, Aparecida de Goiania, Brazil
C*07:1165	KSR175690	PQ329221	Dr Maria Loginova, Kirov, Russia
C*07:1166	KSR103069	PQ329223	Dr Maria Loginova, Kirov, Russia
C*07:1167	HG00052666	PQ310441	Histogenetics, Ossining, NY, USA
C*07:1168	rdkm570144	PP947788	Ms Anastasiia Ananeva, Moscow, Russia
C*07:1169	rdkm852128	PQ062243	Ms Anastasiia Ananeva, Moscow, Russia
C*07:1170	42441056	PQ471514	Dr Romain Ferru‐Clement, Poitiers, France
C*07:1171	HG00055223	PQ414914	Histogenetics, Ossining, NY, USA
C*07:1172	2402593	PQ352479	Dr Mirzokhid Rakhmanov, Freiburg, Germany
C*07:1173	2410499887	PQ588427	Dr Jonathan Visentin, Bordeaux, France
C*08:03:06	HG00055246	PQ535523	Histogenetics, Ossining, NY, USA
C*08:295	42342078	PQ352353	Dr Romain Ferru‐Clement, Poitiers, France
C*08:296	KSR155348, KSR155354	PQ329225	Dr Maria Loginova, Kirov, Russia
C*08:297	rdkm511100	PP947784	Ms Anastasiia Ananeva, Moscow, Russia
C*08:298	rdkm154167	PP974456	Ms Anastasiia Ananeva, Moscow, Russia
C*08:299	HNHLANEW‐15	PQ451109	Dr Zheng Liu, Zhengzhou, China
C*08:300	317841	PQ357060	Dr Raquel Fabreti, Belo Horizonte, Brazil
C*12:02:55	101317	PQ360868	Mr Valery Cheranev, Moscow, Russia
C*12:02:56	HG00055272	PQ594960	Histogenetics, Ossining, NY, USA
C*12:03:01:73	GR‐EVA‐59426	PQ261108	Dr Diamanto Kouniaki, Athens, Greece
C*12:03:93	KSR190697	PQ329227	Dr Maria Loginova, Kirov, Russia
C*12:96:02	169/24	PQ156960	Dr Rajni Chauhan, Gurugram, India
C*12:428N	KSR204911	PQ329226	Dr Maria Loginova, Kirov, Russia
C*12:429	HG00052668	PQ346914	Histogenetics, Ossining, NY, USA
C*12:430	119849	PQ633302	Dr Francisco de Paula Sanchez Gordo, Malaga, Spain
C*14:167	GR‐EVA‐59280	PQ261107	Dr Diamanto Kouniaki, Athens, Greece
C*14:168	NTV_6040581	PQ535549	Dr Georgia Daniela Marcusso, Porto Velho, Brazil
C*15:02:01:67	636/24	PP897958	Dr Rajni Chauhan, Gurugram, India
C*15:02:01:68	NGS1251D3	PQ469238	Dr Meenaskshi Singh, Navi Mumbai, India
C*15:02:01:69Q	2300397	PQ475070	Dr Mirzokhid Rakhmanov, Freiburg, Germany
C*15:290	CMC‐E‐009	PQ261033	Mrs So‐Jung Choi, Seoul, South Korea
C*15:291N	HG00055277	PQ619030	Histogenetics, Ossining, NY, USA
C*16:01:01:42Q	FG 9117	PQ311660	Dr Thibault Pajot, Lille, France
C*16:01:51	42306043	OR130521	Dr Romain Ferru‐Clement, Poitiers, France
C*16:224	HG00055222	PQ414913	Histogenetics, Ossining, NY, USA
C*16:225	HG00055275	PQ594963	Histogenetics, Ossining, NY, USA
C*17:03:01:10	5345146	PP928409	Prof Renata Zunec, Zagreb, Croatia
C*17:82	KSR198800	PQ329228	Dr Maria Loginova, Kirov, Russia
E*01:01:01:65	PSCDA0700	PQ479190	Dr Seik‐Soon Khor, Tokyo, Japan
E*01:01:45	DR1303	PQ412518	Dr Cathi Murphey, San Antonio, TX, USA
E*01:03:02:42	PSCDA0346	PQ479191	Dr Seik‐Soon Khor, Tokyo, Japan
E*01:03:42	PSCDA0957	PQ479189	Dr Seik‐Soon Khor, Tokyo, Japan
F*01:01:01:55	PSCDA0338	PQ329343	Dr Seik‐Soon Khor, Tokyo, Japan
F*01:01:01:56	PSCDA0049	PQ329345	Dr Seik‐Soon Khor, Tokyo, Japan
F*01:01:01:57	PSCDA1012	PQ329347	Dr Seik‐Soon Khor, Tokyo, Japan
F*01:01:01:58	PSCDA0228	PQ329349	Dr Seik‐Soon Khor, Tokyo, Japan
F*01:01:02:16	PSCDA0906	PQ329348	Dr Seik‐Soon Khor, Tokyo, Japan
F*01:14:01:02N	PSCDA0916	PQ329346	Dr Seik‐Soon Khor, Tokyo, Japan
F*01:22	PSCDA1116	PQ329344	Dr Seik‐Soon Khor, Tokyo, Japan
F*01:23	BMD8288	PQ412519	Dr Cathi Murphey, San Antonio, TX, USA
F*01:24	PSCDA0799	PQ479192	Dr Seik‐Soon Khor, Tokyo, Japan
DRB1*01:01:48	41951077	ON062353	Dr Romain Ferru‐Clement, Poitiers, France
DRB1*01:155	JH0152	PQ461649	Dr Maria Bettinotti, Baltimore, MD, USA
DRB1*03:02:07	NTV_5966894	PQ535550	Dr Georgia Daniela Marcusso, Porto Velho, Brazil
DRB1*03:72:02	6740	PQ621718	Dr Dan Zhang, Beijing, China
DRB1*04:01:25	42226124	OR130519	Dr Romain Ferru‐Clement, Poitiers, France
DRB1*04:390	24072663924	PQ130405	Dr Cathi Murphey, San Antonio, TX, USA
DRB1*04:391	ksr117140	PQ240360	Dr Maria Loginova, Kirov, Russia
DRB1*07:01:35	d13863	PQ530372	Dr Cathi Murphey, San Antonio, TX, USA
DRB1*07:60:02	017810779	PQ308712	Dr Xavier Fournel, Saint Etienne, France
DRB1*07:164	KSR161449	PQ240361	Dr Maria Loginova, Kirov, Russia
DRB1*07:165	HG00055238	PQ481500	Histogenetics, Ossining, NY, USA
DRB1*07:166	HG00055302	PQ594968	Histogenetics, Ossining, NY, USA
DRB1*08:132	KSR161427	PQ240362	Dr Maria Loginova, Kirov, Russia
DRB1*08:133	KSR162414	PQ240363	Dr Maria Loginova, Kirov, Russia
DRB1*08:134	MRB2363	PP763746	Dr Eugene Albert, Dolgoprudny, Russian Federation
DRB1*09:01:16	90458134234546	PQ304399	Dr Antonio Balas, Madrid, Spain
DRB1*09:01:17	117916	PQ349321	Dr Francisco de Paula Sanchez Gordo, Malaga, Spain
DRB1*09:61N	24817626	PQ383846	Dr Luis A. Marin, Albacete, Spain
DRB1*11:04:25	MRB3150	PP763748	Dr Eugene Albert, Dolgoprudny, Russian Federation
DRB1*11:339	KSR189637	PQ240364	Dr Maria Loginova, Kirov, Russia
DRB1*11:340	2402560	PQ346316	Dr Paul Rouzaire, Clermont‐Ferrand, France
DRB1*12:114	HG00055236	PQ414915	Histogenetics, Ossining, NY, USA
DRB1*13:01:44	M8570	PQ323363	Dr Thibault Pajot, Lille, France
DRB1*13:364	KSR175857	PQ240365	Dr Maria Loginova, Kirov, Russia
DRB1*13:365	1123076801	PQ381335	Mr Gerald Bertrand, Rennes Cedex, France
DRB1*13:366	TZX‐1	PQ633268	Dr Cheng Yan Fan, Beijing, China
DRB1*13:367N	HG00055306	PQ655365	Histogenetics, Ossining, NY, USA
DRB1*14:54:14	JH0151, JH0151B	PQ439177	Dr Maria Bettinotti, Baltimore, MD, USA
DRB1*14:270	NRCH00045	PQ178956	Mr Evgeny Leonov, Moscow, Russia
DRB1*14:271	42413038	PQ268524	Dr Romain Ferru‐Clement, Poitiers, France
DRB1*14:272	NTV_6102280	PQ535551	Dr Georgia Daniela Marcusso, Porto Velho, Brazil
DRB1*14:273	HG00055303	PQ619027	Histogenetics, Ossining, NY, USA
DRB1*15:01:01:33	204_00505	PP072297	Dr Li Zihang, Xi'an, China
DRB1*15:01:53	42412003	PQ268523	Dr Romain Ferru‐Clement, Poitiers, France
DRB1*15:234	HG00055237	PQ414916	Histogenetics, Ossining, NY, USA
DRB1*15:235	HG00055305	PQ655364	Histogenetics, Ossining, NY, USA
DRB3*01:131	42425051	WS100923	Dr Romain Ferru‐Clement, Poitiers, France
DRB3*01:132N	5088518	PQ360796	Prof Renata Zunec, Zagreb, Croatia
DRB3*01:133N	5347033	PQ360797	Prof Renata Zunec, Zagreb, Croatia
DRB3*02:02:01:18	S7632DRB	PQ212890	Dr Justine Schmitt, Liege, Belgium
DRB3*02:213	172317	PQ179044	Dr Charlene Bouthemy, Toulouse, France
DRB3*02:214N	017035198	PQ295903	Dr Margot Lepage, Decines‐Charpieu, France
DRB3*02:215	JH0149	PQ227097	Dr Maria Bettinotti, Baltimore, MD, USA
DRB3*03:77	RP‐284980	PQ311669	Dr Neifi Hassan Saloum Deghaide, Ribeirao Preto, Brazil
DRB3*03:78	RP‐289498	PQ621782	Dr Neifi Hassan Saloum Deghaide, Ribeirao Preto, Brazil
DRB4*01:03:01:21	201‐00380	PP072298	Dr Li Zihang, Xi'an, China
DRB4*01:03:01:22	201_00754	PP955962	Ms Yuanli Zhao, Xi'an, China
DRB4*01:03:01:23	201‐00605	PP961285	Ms Yuanli Zhao, Xi'an, China
DRB4*01:03:01:24	201‐00558	PQ539411	Ms Yuanli Zhao, Xi'an, China
DRB4*01:03:01:25	204‐01654	PP955967	Dr Li Zihang, Xi'an, China
DRB4*01:03:01:26	201‐00594	PP955965	Ms Yuanli Zhao, Xi'an, China
DRB4*01:03:02:03	201‐00558S	PP955970	Ms Yuanli Zhao, Xi'an, China
DRB4*01:189	NGR0109	PQ276757	Dr Laura Bungener, Groningen, The Netherlands
DRB4*01:190	42426018	PQ553181	Dr Romain Ferru‐Clement, Poitiers, France
DRB5*01:01:01:08	201‐00009	PQ559719	Ms Yuanli Zhao, Xi'an, China
DRB5*01:01:01:09	201‐00326	PQ559720	Ms Yuanli Zhao, Xi'an, China
DRB5*01:01:01:10	201‐00176	PQ476166	Dr Li Zihang, Xi'an, China
DRB5*01:01:01:11	22WB00043	PQ559723	Ms Yuanli Zhao, Xi'an, China
DRB5*01:02:05	Amiens15	PQ368758	Dr Nicolas Guillaume, Amiens, France
DRB5*01:143	241822	PQ586434	Dr Taba Kheradmand, Long Island City, NY, USA
DQA1*01:01:14	103926318	PQ304398	Dr Antonio Balas, Madrid, Spain
DQA1*01:10:02	42133089	PQ310666	Dr Romain Ferru‐Clement, Poitiers, France
DQA1*01:19:01:02	SMC015	LC835498	Dr Eunsuk Kang, Seoul, South Korea
DQA1*01:173	24‐0651	PQ111547	Dr M. Rosa Moya‐Quiles, El Palmar, Spain
DQA1*01:174	017909139	PQ273915	Dr Margot Lepage, Decines‐Charpieu, France
DQA1*01:175	YUMC019	LC848301	Dr YoonHee Park, Seoul, South Korea
DQA1*01:176	YUMC020	LC848303	Dr YoonHee Park, Seoul, South Korea
DQA1*01:177	RP‐289505	PQ590133	Dr Neifi Hassan Saloum Deghaide, Ribeirao Preto, Brazil
DQA1*01:178	HG00055291	PQ655360	Histogenetics, Ossining, NY, USA
DQA1*01:179	HG00055289	PQ594965	Histogenetics, Ossining, NY, USA
DQA1*02:01:25	HG00055290	PQ594966	Histogenetics, Ossining, NY, USA
DQA1*02:48	HG00052682	PQ346920	Histogenetics, Ossining, NY, USA
DQA1*02:49	509655	PP955178	Mr Alessandro Pirri, Curitiba, Brazil
DQA1*03:01:01:07	509831	PQ257003	Mr Alessandro Pirri, Curitiba, Brazil
DQA1*03:01:18	117767	PQ196789	Dr Francisco de Paula Sanchez Gordo, Malaga, Spain
DQA1*03:03:10	RP‐284218	PQ362996	Dr Neifi Hassan Saloum Deghaide, Ribeirao Preto, Brazil
DQA1*03:82	24‐1903	PQ111548	Dr M. Rosa Moya‐Quiles, El Palmar, Spain
DQA1*03:83	HG00055229	PQ497701	Histogenetics, Ossining, NY, USA
DQA1*03:84	HG00055230	PQ497702	Histogenetics, Ossining, NY, USA
DQA1*04:26	RP‐287923	PQ634388	Dr Neifi Hassan Saloum Deghaide, Ribeirao Preto, Brazil
DQA1*05:01:20	509988	PQ351808	Mr Alessandro Pirri, Curitiba, Brazil
DQA1*05:118	40145867	PQ304400	Dr Antonio Balas, Madrid, Spain
DQA1*05:119	E000724045494	PQ512829	Dr Dolores Planelles, Valencia, Spain
DQA1*05:120	65241086544	PQ411188	Mr Gerald Bertrand, Rennes Cedex, France
DQA1*06:12	42417021	PQ268522	Dr Romain Ferru‐Clement, Poitiers, France
DQB1*05:01:01:40	201‐00606	PQ241145	Ms Yuanli Zhao, Xi'an, China
DQB1*05:03:38	SZ‐233	PQ111024	Dr Hong‐Yan Zou, Shenzhen, China
DQB1*05:311:02	FG9221	PQ323365	Dr Thibault Pajot, Lille, France
DQB1*05:353	735866	PQ276067	Dr Luis A. Marin, Albacete, Spain
DQB1*05:354	FG 9123	PQ311659	Dr Thibault Pajot, Lille, France
DQB1*05:355	A8115	PQ346949	Dr Thibault Pajot, Lille, France
DQB1*05:356	FG 7320	PQ394572	Dr Vincent Elsermans, Lille cedex, France
DQB1*05:357	H7481	PQ349975	Dr Vincent Elsermans, Lille cedex, France
DQB1*06:02:01:42	201_00991	PQ241149	Ms Yuanli Zhao, Xi'an, China
DQB1*06:02:65	RMU2827	PQ360874	Mr Valery Cheranev, Moscow, Russia
DQB1*06:520	NCY_IMT_01	PP235384	Dr Adele Dhuyser, Vandoeuvre Les Nancy, France
DQB1*06:521	RP‐284679	PQ311668	Dr Neifi Hassan Saloum Deghaide, Ribeirao Preto, Brazil
DQB1*06:522	65220256436	PQ268832	Mr Gerald Bertrand, Rennes Cedex, France
DQB1*06:523	240502DQBv	PP889576	Dr Zeying Du, Jacksonville, FL, USA
DQB1*06:524	6856	PQ621719	Dr Dan Zhang, Beijing, China
DQB1*06:525	K_6054	PQ634766	Dr Samir Agarwal, New Delhi, India
DQB1*02:01:01:26	71618519	PQ451430	Dr Luis A. Marin, Albacete, Spain
DQB1*02:01:01:27	201‐00609	PQ241139	Ms Yuanli Zhao, Xi'an, China
DQB1*02:01:01:28	201_00749	PQ241140	Ms Yuanli Zhao, Xi'an, China
DQB1*02:01:01:29	201_00749S	PQ241141	Ms Yuanli Zhao, Xi'an, China
DQB1*02:02:01:24	201‐00601	PQ241138	Ms Yuanli Zhao, Xi'an, China
DQB1*02:02:01:25	201‐00377	PQ241137	Ms Yuanli Zhao, Xi'an, China
DQB1*02:02:31	NTV_6104090	PQ535552	Dr Georgia Daniela Marcusso, Porto Velho, Brazil
DQB1*02:239	9000624500047091	PP599022	Prof Faming Zhu, Hangzhou, China
DQB1*02:240	42405101	PQ268521	Dr Romain Ferru‐Clement, Poitiers, France
DQB1*02:241	016572700	PQ280742	Dr Margot Lepage, Decines‐Charpieu, France
DQB1*02:242	65220403272	PQ381328	Mr Gerald Bertrand, Rennes Cedex, France
DQB1*02:243	6523132473‐	PQ381336	Mr Gerald Bertrand, Rennes Cedex, France
DQB1*02:244	24224039	PQ564472	Dr Daniel Fuerst, Ulm, Germany
DQB1*02:245	HG00055299	PQ619029	Histogenetics, Ossining, NY, USA
DQB1*03:01:01:71	201‐00326S	PQ476168	Ms Yuanli Zhao, Xi'an, China
DQB1*03:01:01:72	HR5351022	PQ567081	Prof Renata Zunec, Zagreb, Croatia
DQB1*03:01:01:73	04721226	PQ359805	Dr Antonio Balas, Madrid, Spain
DQB1*03:02:01:30	204‐01703	PQ241136	Ms Yuanli Zhao, Xi'an, China
DQB1*03:02:47	rdkm415148	PQ130284	Ms Anastasiia Ananeva, Moscow, Russia
DQB1*03:566	NCY_EGD_01	PP566109	Dr Adele Dhuyser, Vandoeuvre Les Nancy, France
DQB1*03:567	900612290222726	PP599023	Prof Faming Zhu, Hangzhou, China
DQB1*03:568	24‐1122	PQ559523	Assoc Prof Ahmed Mostafa, Saskatoon, Canada
DQB1*03:569	JH0122	PP504566	Dr Maria Bettinotti, Baltimore, MD, USA
DQB1*03:570	BMD4198	PQ241317	Dr Cathi Murphey, San Antonio, TX, USA
DQB1*03:571	24‐2136	PQ394576	Dr M. Rosa Moya‐Quiles, El Palmar, Spain
DQB1*03:572	CX‐24	PQ241482	Dr Zhangxiang Wanyan, Kunming, China
DPA1*01:03:01:96	204_00707	PP961283	Ms Yuanli Zhao, Xi'an, China
DPA1*01:03:61	2402147	PP986398	Dr Paul Rouzaire, Clermont‐Ferrand, France
DPA1*01:04:01:03	24404726	PQ276068	Dr Luis A. Marin, Albacete, Spain
DPA1*01:155:01:03	2022030506	PP869622	Mr Jobson Nascimento, Recife, Brazil
DPA1*01:222	NRCH00043	PP982819	Mr Evgeny Leonov, Moscow, Russia
DPA1*01:223	JH0144	PQ196105	Dr Maria Bettinotti, Baltimore, MD, USA
DPA1*01:224	HS26594	PQ273166	Dr Cathi Murphey, San Antonio, TX, USA
DPA1*01:225	NGR0108	PQ276758	Dr Laura Bungener, Groningen, The Netherlands
DPA1*01:226	24220821	PQ381296	Dr Daniel Fuerst, Ulm, Germany
DPA1*01:227	HG00055208	PQ369424	Histogenetics, Ossining, NY, USA
DPA1*01:228	24819258	PQ479078	Dr Luis A. Marin, Albacete, Spain
DPA1*01:229	OT1866	PQ463967	Dr Cathi Murphey, San Antonio, TX, USA
DPA1*01:230	119314	PQ505995	Dr Francisco de Paula Sanchez Gordo, Malaga, Spain
DPA1*01:231	W1648423	PQ417931	Dr Prabhakar Putheti, Valhalla, USA
DPA1*02:01:01:40	24819233	PQ383848	Dr Luis A. Marin, Albacete, Spain
DPA1*02:01:01:41	24404620	PQ479080	Dr Luis A. Marin, Albacete, Spain
DPA1*02:01:32	RP‐285305	PQ311670	Dr Neifi Hassan Saloum Deghaide, Ribeirao Preto, Brazil
DPA1*02:01:33	42430010	PQ352354	Dr Romain Ferru‐Clement, Poitiers, France
DPA1*02:01:34	23‐0352	PQ550135	Assoc Prof Ahmed Mostafa, Saskatoon, Canada
DPA1*02:01:35	GLAS95693	PQ565818	Dr Claire Walker, Glasgow, UK
DPA1*02:02:02:21	201_01197	PP961277	Ms Yuanli Zhao, Xi'an, China
DPA1*02:147N	201‐00009	PP083722	Dr Li Zihang, Xi'an, China
DPA1*02:148N	HG00052670	PQ310447	Histogenetics, Ossining, NY, USA
DPA1*02:149	HG00052675	PQ346919	Histogenetics, Ossining, NY, USA
DPA1*03:20Q	1222‐28433	PQ275501	Dr Yanping Huang, Philadelphia, USA
DPB1*04:01:93	1179658	PQ196790	Dr Francisco de Paula Sanchez Gordo, Malaga, Spain
DPB1*04:01:94	118949	PQ462263	Dr Francisco de Paula Sanchez Gordo, Malaga, Spain
DPB1*13:01:09	41836106	ON062361	Dr Romain Ferru‐Clement, Poitiers, France
DPB1*17:01:01:10	5344883	PQ409322	Prof Renata Zunec, Zagreb, Croatia
DPB1*133:01:02	JH0150	PQ429077	Dr Maria Bettinotti, Baltimore, MD, USA
DPB1*1730:01	2024TB13231	PQ161094	Dr Keming Du, Shanghai, China
DPB1*1731:01	DR844	PQ273165	Dr Cathi Murphey, San Antonio, TX, USA
DPB1*1732:01	424290	PQ281495	Mrs Tamara Passos, Salvador, Brazil
DPB1*1733:01	021454744	PQ280741	Dr Margot Lepage, Decines‐Charpieu, France
DPB1*1734:01	202317906	PQ305757	Dr Amalia Tejeda Velarde, Salamanca, Spain
DPB1*1735:01	42433062	PQ310668	Dr Romain Ferru‐Clement, Poitiers, France
DPB1*1736:01	LIB_6180188	PQ316046	Ms Anaregina Araujo, Teresina, Brazil
DPB1*1737:01	317027	PQ181521	Dr Raquel Fabreti, Belo Horizonte, Brazil
DPB1*1738:01	81350694	PQ310554	Dr Daniel Fuerst, Ulm, Germany
DPB1*1739:01	65220733837	PQ381227	Mr Gerald Bertrand, Rennes Cedex, France
DPB1*1740:01	HG00052677	PQ310449	Histogenetics, Ossining, NY, USA
DPB1*1741:01	HG00052679	PQ346924	Histogenetics, Ossining, NY, USA
DPB1*1742:01	022033131	PQ286037	Mrs Ornella Senoussi, La Tronche, France
DPB1*1743:01	022099018	PQ286038	Mrs Ornella Senoussi, La Tronche, France
DPB1*1744:01	d11508	PQ273164	Dr Cathi Murphey, San Antonio, TX, USA
DPB1*1744:01	45640	OR474513	Dr Yanping Huang, Philadelphia, USA
DPB1*1745:01	118939	PQ449570	Dr Francisco de Paula Sanchez Gordo, Malaga, Spain
DPB1*1746:01	HG00055248	PQ535520	Histogenetics, Ossining, NY, USA
DPB1*1747:01	HG00055249	PQ535521	Histogenetics, Ossining, NY, USA
DPB1*1748:01	24‐1095	PQ550137	Assoc Prof Ahmed Mostafa, Saskatoon, Canada
DPB1*1749:01	d13650	PQ309533	Dr Cathi Murphey, San Antonio, TX, USA
DPB1*1750:01	P15449‐1	PQ535698	Prof Faming Zhu, Hangzhou, China
DPB1*1751:01	HG00055286	PQ619039	Histogenetics, Ossining, NY, USA
DPB1*1752:01	HG00055282	PQ594970	Histogenetics, Ossining, NY, USA
DPB1*1753:01	HG00055283	PQ594971	Histogenetics, Ossining, NY, USA
DPB1*1754:01	HG00055287	PQ619040	Histogenetics, Ossining, NY, USA
DPB1*1755:01	HG00055288	PQ655356	Histogenetics, Ossining, NY, USA
DPB1*1756:01	HG00055285	PQ594973	Histogenetics, Ossining, NY, USA
DPB1*1757:01	HG00055284	PQ594972	Histogenetics, Ossining, NY, USA
DMA*01:01:01:43	AN303513	OZ184115	Anthony Nolan Research Institute, London, UK
DMA*01:01:03	AN303514	OZ184116	Anthony Nolan Research Institute, London, UK
DMB*01:01:01:52	DKMS‐LSL_ID19880681, DKMS‐LSL_ID19880124	OZ177759, OZ177760	DKMS Life Sciences Lab., Dresden, Germany
DMB*01:01:01:53	AN303521	OZ184123	Anthony Nolan Research Institute, London, UK
DMB*01:01:01:54	AN303523	OZ184125	Anthony Nolan Research Institute, London, UK
DMB*01:01:01:55	AN303519	OZ184121	Anthony Nolan Research Institute, London, UK
DMB*01:01:01:56	AN303520	OZ184122	Anthony Nolan Research Institute, London, UK
DMB*01:01:01:57	AN303515, AN303516	OZ184117, OZ184118	Anthony Nolan Research Institute, London, UK
DMB*01:01:01:58	AN303524	OZ184126	Anthony Nolan Research Institute, London, UK
DMB*01:01:01:59	AN303518	OZ184120	Anthony Nolan Research Institute, London, UK
DMB*01:01:01:60	AN303517	OZ184119	Anthony Nolan Research Institute, London, UK
DMB*01:03:01:20	AN303525	OZ184127	Anthony Nolan Research Institute, London, UK
DMB*01:03:01:21	AN303526	OZ184128	Anthony Nolan Research Institute, London, UK
DMB*01:03:01:22	AN302579, AN303347, AN303522	OZ184112, OZ184114, OZ184124	Anthony Nolan Research Institute, London, UK
DMB*01:03:01:23	AN303527	OZ184129	Anthony Nolan Research Institute, London, UK
DMB*01:07:01:05	AN303528	OZ184130	Anthony Nolan Research Institute, London, UK
DMB*01:07:01:06	AN303529, AN303530	OZ184131, OZ184132	Anthony Nolan Research Institute, London, UK
DOA*01:01:01:13	AN303531	OZ184133	Anthony Nolan Research Institute, London, UK
DOA*01:01:02:56	AN303535	OZ184137	Anthony Nolan Research Institute, London, UK
DOA*01:01:02:57	AN303536	OZ184138	Anthony Nolan Research Institute, London, UK
DOA*01:01:02:58	AN303533	OZ184135	Anthony Nolan Research Institute, London, UK
DOA*01:01:02:59	AN303534	OZ184136	Anthony Nolan Research Institute, London, UK
DOA*01:01:02:60	AN303532	OZ184134	Anthony Nolan Research Institute, London, UK
DOA*01:01:03:07	AN300743, AN303537	OZ184110, OZ184139	Anthony Nolan Research Institute, London, UK
DOA*01:01:04:14	AN300758	OZ184111	Anthony Nolan Research Institute, London, UK
DOB*01:01:01:31	AN303541	OZ184143	Anthony Nolan Research Institute, London, UK
DOB*01:01:01:32	AN303539	OZ184141	Anthony Nolan Research Institute, London, UK
DOB*01:01:01:33	AN303540	OZ184142	Anthony Nolan Research Institute, London, UK
DOB*01:01:01:34	AN303538	OZ184140	Anthony Nolan Research Institute, London, UK
DOB*01:01:03:11	AN303542	OZ184144	Anthony Nolan Research Institute, London, UK
DOB*01:01:05:02	AN303012, AN303543	OZ184113, OZ184145	Anthony Nolan Research Institute, London, UK
DOB*01:02:01:04	AN303544	OZ184146	Anthony Nolan Research Institute, London, UK
DOB*01:03:01:03	AN303545	OZ184147	Anthony Nolan Research Institute, London, UK
DOB*01:10:01:02	AN303546, AN303547	OZ184148, OZ184149	Anthony Nolan Research Institute, London, UK
MICA*001:01:02	CRB	OZ179402	Anthony Nolan Research Institute, London, UK
MICA*002:01:26	KRC‐110	OZ179572	Anthony Nolan Research Institute, London, UK
MICA*002:01:27	RSA‐ND	OZ179673	Anthony Nolan Research Institute, London, UK
MICA*002:01:28	OZB	OZ179277	Anthony Nolan Research Institute, London, UK
MICA*002:01:29	HGOM	OZ179504	Anthony Nolan Research Institute, London, UK
MICA*008:04:10	LIE	OZ179595	Anthony Nolan Research Institute, London, UK
MICA*008:04:11	GUA‐ND	OZ179490	Anthony Nolan Research Institute, London, UK
MICA*008:04:12	PAR	OZ179647	Anthony Nolan Research Institute, London, UK
MICA*009:01:14	LIE	OZ179596	Anthony Nolan Research Institute, London, UK
MICA*009:02:07	UCLA213	OZ198365	Anthony Nolan Research Institute, London, UK
MICA*010:01:15	A2393	PP420217	Dr Meenaskshi Singh, Navi Mumbai, India
MICA*010:01:16	HAS‐15	OZ179496	Anthony Nolan Research Institute, London, UK
MICA*010:01:17	UCLA209	OZ198363	Anthony Nolan Research Institute, London, UK
MICA*010:01:18	UCLA215	OZ198367	Anthony Nolan Research Institute, London, UK
MICA*012:01:08	UCLA215	OZ198368	Anthony Nolan Research Institute, London, UK
MICA*018:01:13	UCLA208	OZ198362	Anthony Nolan Research Institute, London, UK
MICA*019:01:10	RSA‐ND	OZ179674	Anthony Nolan Research Institute, London, UK
MICA*019:01:11	UCLA214	OZ198366	Anthony Nolan Research Institute, London, UK
MICB*002:01:39	RSA‐ND	OZ179675	Anthony Nolan Research Institute, London, UK
MICB*004:01:30	LK707	OZ179601	Anthony Nolan Research Institute, London, UK
MICB*004:01:31	D4_GS_D4	PQ281434	Dr Gaurav Sharma, Chandigarh, India
MICB*004:01:32	R11_GS_11	PQ296825	Dr Gaurav Sharma, Chandigarh, India
MICB*004:01:33	R3_GS_3	PQ296823	Dr Gaurav Sharma, Chandigarh, India
MICB*005:02:53	35020	OZ179293	Anthony Nolan Research Institute, London, UK
MICB*005:02:54	35841	OZ179298	Anthony Nolan Research Institute, London, UK
MICB*005:02:55	32511	OZ179289	Anthony Nolan Research Institute, London, UK
MICB*005:02:56	32367	OZ179286	Anthony Nolan Research Institute, London, UK
MICB*005:02:57	BER, BH, LBF, TL, XLI‐ND	OZ179331, OZ179335, OZ179588, OZ179714, OZ179750	Anthony Nolan Research Institute, London, UK
MICB*005:02:58	C073	OZ179371	Anthony Nolan Research Institute, London, UK
MICB*005:02:59	R11_GS_11	PQ296826	Dr Gaurav Sharma, Chandigarh, India
MICB*005:03:09	LIE	OZ179597	Anthony Nolan Research Institute, London, UK
MICB*008:01:19	32511	OZ179290	Anthony Nolan Research Institute, London, UK
MICB*008:01:20	35020	OZ179294	Anthony Nolan Research Institute, London, UK

**TABLE 2:** Confirmatory sequences.

HLA allele	Cell identification	Accession number	Submitting author
A*01:26	NT00736	PQ046249	Dr Jennifer Ng, Rockville, MD, USA
A*02:22:02	NT01044	PQ046256	Dr Jennifer Ng, Rockville, MD, USA
A*02:84	NT00747	PQ046250	Dr Jennifer Ng, Rockville, MD, USA
A*02:147	NT00793	PQ015335	Dr Jennifer Ng, Rockville, MD, USA
A*02:178	NT01055	PQ046253	Dr Jennifer Ng, Rockville, MD, USA
A*02:350N	KSR189936	PQ304146	Dr Maria Loginova, Kirov, Russia
A*02:664	509738	PQ483445	Mr Alessandro Pirri, Curitiba, Brazil
A*02:664	503410	PQ463963	Mr Alessandro Pirri, Curitiba, Brazil
A*02:681	119436	PQ505997	Dr Francisco de Paula Sanchez Gordo, Malaga, Spain
A*02:812	LIB_6222708	PQ416619	Ms Anaregina Araujo, Teresina, Brazil
A*02:950	LIB_6239996	PQ416621	Ms Anaregina Araujo, Teresina, Brazil
A*03:16	NT01196	PQ064534	Dr Jennifer Ng, Rockville, MD, USA
A*11:19	NT00745	PQ046251	Dr Jennifer Ng, Rockville, MD, USA
A*11:20	NT01054	PQ046255	Dr Jennifer Ng, Rockville, MD, USA
A*11:24:01	NT01197	PQ064535	Dr Jennifer Ng, Rockville, MD, USA
A*23:144	2492587	PQ492170	Mrs Ana Lucia Rodrigues, Aparecida de Goiania, Brazil
A*24:02:06	NT01165	PQ064530	Dr Jennifer Ng, Rockville, MD, USA
A*24:24	NT01164	PQ064532	Dr Jennifer Ng, Rockville, MD, USA
A*24:47	NT01158	PQ064533	Dr Jennifer Ng, Rockville, MD, USA
A*24:55	NT00620	PQ064537	Dr Jennifer Ng, Rockville, MD, USA
A*24:459:02	LIB_6093209	PQ240517	Ms Anaregina Araujo, Teresina, Brazil
A*24:570	501943	PQ412539	Mr Alessandro Pirri, Curitiba, Brazil
A*24:570	LIB_6245201	PQ416620	Ms Anaregina Araujo, Teresina, Brazil
A*26:248	HG00055212, HG00055213	PQ414910, PQ414911	Histogenetics, Ossining, NY, USA
A*30:02:33	1704647	PQ349843	Prof Maria Gerbase‐DeLima, Sao Paulo, Brazil
A*30:13	NT00513	PQ064536	Dr Jennifer Ng, Rockville, MD, USA
A*30:184Q	2024‐30184	PQ308085	Mrs Silvia Ulrich, Graz, Austria
A*30:213	118662	PQ280323	Dr Francisco de Paula Sanchez Gordo, Malaga, Spain
A*31:21	NT00738	PQ015334	Dr Jennifer Ng, Rockville, MD, USA
A*31:177	506375	PQ589720	Mr Alessandro Pirri, Curitiba, Brazil
A*31:182	1012189596	PQ374119	Dr Thibaut Gervais, Brussels, Belgium
A*32:01:14	AN32821111	PQ274558	Anthony Nolan Research Institute, London, UK
A*33:59	NGR0112A	PQ276761	Dr Laura Bungener, Groningen, The Netherlands
A*36:15	RP‐287466	PQ570119	Dr Neifi Hassan Saloum Deghaide, Ribeirao Preto, Brazil
A*68:01:28	118461	PQ196791	Dr Francisco de Paula Sanchez Gordo, Malaga, Spain
A*68:48	NT01063	PQ046254	Dr Jennifer Ng, Rockville, MD, USA
A*68:137	NT01234	PQ064538	Dr Jennifer Ng, Rockville, MD, USA
A*68:322	HD8110	PQ323367	Dr Thibault Pajot, Lille, France
A*74:01:08	LIB_5887620	PQ416622	Ms Anaregina Araujo, Teresina, Brazil
A*74:05	NT01065	PQ046252	Dr Jennifer Ng, Rockville, MD, USA
B*07:02:21	KSR129952	PQ304147	Dr Maria Loginova, Kirov, Russia
B*07:327	510074	PQ463965	Mr Alessandro Pirri, Curitiba, Brazil
B*07:405	118588	PQ280322	Dr Francisco de Paula Sanchez Gordo, Malaga, Spain
B*07:475	DKMS‐LSL_ID22043619	OZ174415	DKMS Life Sciences Lab., Dresden, Germany
B*07:488	DKMS‐LSL_ID19998795	OZ174396	DKMS Life Sciences Lab., Dresden, Germany
B*08:93	NGR0110	PQ276759	Dr Laura Bungener, Groningen, The Netherlands
B*08:152	KSR181797	PQ304150	Dr Maria Loginova, Kirov, Russia
B*08:285	DKMS‐LSL_ID20766331	OZ174384	DKMS Life Sciences Lab., Dresden, Germany
B*08:315	DKMS‐LSL_ID21264177	OZ174421	DKMS Life Sciences Lab., Dresden, Germany
B*14:02:15	KSR140622	PQ304148	Dr Maria Loginova, Kirov, Russia
B*15:18:02	KSR1409	PQ304153	Dr Maria Loginova, Kirov, Russia
B*15:683	DKMS‐LSL_ID20011336	OZ174398	DKMS Life Sciences Lab., Dresden, Germany
B*15:704	DKMS‐LSL_ID20959346	OZ174431	DKMS Life Sciences Lab., Dresden, Germany
B*18:01:53	506917	PQ621278	Mr Alessandro Pirri, Curitiba, Brazil
B*18:03:01:01	DKMS‐LSL_ID20627355	OZ174437	DKMS Life Sciences Lab., Dresden, Germany
B*18:31	KSR197249	PQ304151	Dr Maria Loginova, Kirov, Russia
B*18:36	MC091924B18	PQ428923	Dr Zeying Du, Jacksonville, FL, USA
B*18:224	507576	PQ621277	Mr Alessandro Pirri, Curitiba, Brazil
B*27:110	503683	PQ483447	Mr Alessandro Pirri, Curitiba, Brazil
B*35:01:05	DKMS‐LSL_ID20118418	OZ174381	DKMS Life Sciences Lab., Dresden, Germany
B*35:01:79	HG00055245	PQ535513	Histogenetics, Ossining, NY, USA
B*35:02:23	DKMS‐LSL_ID21185023	OZ174426	DKMS Life Sciences Lab., Dresden, Germany
B*35:03:38	DKMS‐LSL_ID20837796	OZ174433	DKMS Life Sciences Lab., Dresden, Germany
B*35:82:02	DKMS‐LSL_ID19960383	OZ174395	DKMS Life Sciences Lab., Dresden, Germany
B*35:535	DKMS‐LSL_ID20709592	OZ174435	DKMS Life Sciences Lab., Dresden, Germany
B*39:09:01:03	DKMS‐LSL_ID20709592	OZ174436	DKMS Life Sciences Lab., Dresden, Germany
B*39:130	THC69	PQ336878	Prof Wei Tian, Changsha, China
B*39:210	DKMS‐LSL_ID20337580	OZ174441	DKMS Life Sciences Lab., Dresden, Germany
B*40:02:01:08	DKMS‐LSL_ID21208194	OZ174422	DKMS Life Sciences Lab., Dresden, Germany
B*40:49	24224456	PQ583039	Dr Daniel Fuerst, Ulm, Germany
B*40:105	2469026	PQ282131	Mrs Ana Lucia Rodrigues, Aparecida de Goiania, Brazil
B*40:173	KSR202919	PQ304152	Dr Maria Loginova, Kirov, Russia
B*40:186:02	SYNPC211	KU598668	Prof Wei Tian, Changsha, China
B*40:298:02	TYNPC27	MF578382	Prof Wei Tian, Changsha, China
B*40:549	DKMS‐LSL_ID21867308	OZ174416	DKMS Life Sciences Lab., Dresden, Germany
B*41:02:01:01	DKMS‐LSL_ID20470913	OZ174439	DKMS Life Sciences Lab., Dresden, Germany
B*44:54	LIB_5908611	PQ559634	Ms Anaregina Araujo, Teresina, Brazil
B*46:01:25	TIM36A	PQ336879	Prof Wei Tian, Changsha, China
B*50:26	ID003400314093	PQ631131	Miss Sriprapai Khanuntong, Bangkok, Thailand
B*51:01:87	118222	PQ196788	Dr Francisco de Paula Sanchez Gordo, Malaga, Spain
B*51:20	24277T0078	PQ595901	Ms Sarah Peacock, Cambridge, UK
B*51:224	119975	PQ633303	Dr Francisco de Paula Sanchez Gordo, Malaga, Spain
B*51:349	DKMS‐LSL_ID20026064	OZ174399	DKMS Life Sciences Lab., Dresden, Germany
B*51:404	DKMS‐LSL_ID20491272	OZ177834	DKMS Life Sciences Lab., Dresden, Germany
B*51:409N	9000624502480093	PQ535694	Prof Faming Zhu, Hangzhou, China
B*51:409N	Amiens14	PQ247708	Dr Nicolas Guillaume, Amiens, France
B*52:119	DKMS‐LSL_ID20900748	OZ174432	DKMS Life Sciences Lab., Dresden, Germany
B*55:07	KSR179029	PQ304149	Dr Maria Loginova, Kirov, Russia
B*58:156	DKMS‐LSL_ID20141841	OZ174412	DKMS Life Sciences Lab., Dresden, Germany
C*01:274	2023071879	MZ714558	Dr Mingrui Huo, Beijing, China
C*02:189	119616	PQ553454	Dr Francisco de Paula Sanchez Gordo, Malaga, Spain
C*02:214	119263	PQ462265	Dr Francisco de Paula Sanchez Gordo, Malaga, Spain
C*02:231	2407017369	PQ356327	Dr Francesco Ingrassia, Palermo, Italy
C*02:249	KSR174790	PQ240368	Dr Maria Loginova, Kirov, Russia
C*03:04:01:22	5343623	PP928407	Prof Renata Zunec, Zagreb, Croatia
C*03:657	KBNUH005	LC849217	Dr Dongil Won, Daegu, South Korea
C*03:689	2023041003	MZ714558	Dr Mingrui Huo, Beijing, China
C*04:532	KSR160866	PQ240372	Dr Maria Loginova, Kirov, Russia
C*05:01:01:86	NGS946	PQ308699	Dr Meenaskshi Singh, Navi Mumbai, India
C*05:01:82	rdkm577163	PP947786	Ms Anastasiia Ananeva, Moscow, Russia
C*06:73	504661	PQ586427	Mr Alessandro Pirri, Curitiba, Brazil
C*06:104	KSR142978	PQ304154	Dr Maria Loginova, Kirov, Russia
C*07:02:156	KSR170213	PQ329222	Dr Maria Loginova, Kirov, Russia
C*07:107	24819828	PQ530046	Dr Luis A. Marin, Albacete, Spain
C*07:365	504878	PQ586426	Mr Alessandro Pirri, Curitiba, Brazil
C*07:373	LIB_6192023	PQ425360	Ms Anaregina Araujo, Teresina, Brazil
C*07:528	118728	PQ349322	Dr Francisco de Paula Sanchez Gordo, Malaga, Spain
C*07:1022	501680	PQ412538	Mr Alessandro Pirri, Curitiba, Brazil
C*08:01:01:08	24404622	PQ479079	Dr Luis A. Marin, Albacete, Spain
C*08:16:01	KSR202922	PQ304155	Dr Maria Loginova, Kirov, Russia
C*15:05:21	FG9642	PQ133119	Dr Vincent Elsermans, Lille cedex, France
C*15:253Q	501315	PQ394278	Mr Alessandro Pirri, Curitiba, Brazil
C*15:261	LIB_6177267	PQ425361	Ms Anaregina Araujo, Teresina, Brazil
C*16:212	501636	PQ483446	Mr Alessandro Pirri, Curitiba, Brazil
C*16:221	HG00055273	PQ594961	Histogenetics, Ossining, NY, USA
E*01:01:01:11	PSCDA2076	PQ317185	Dr Seik‐Soon Khor, Tokyo, Japan
E*01:01:01:20	PSCDA0359	PQ317186	Dr Seik‐Soon Khor, Tokyo, Japan
E*01:03:02:27	PSCDA0874	PQ317183	Dr Seik‐Soon Khor, Tokyo, Japan
E*01:03:02:32	PSCDA0229	PQ317184	Dr Seik‐Soon Khor, Tokyo, Japan
F*01:01:02:07	PMG075	OZ178773	Anthony Nolan Research Institute, London, UK
DRA*01:05	PSCDA0821	PP616718	Dr Seik‐Soon Khor, Tokyo, Japan
DRB1*10:01:18	KSR190640	PQ304156	Dr Maria Loginova, Kirov, Russia
DRB1*12:110	AMC‐E‐009	PQ015209	Mrs So‐Jung Choi, Seoul, South Korea
DRB1*13:60	502151	PQ412540	Mr Alessandro Pirri, Curitiba, Brazil
DRB1*13:319N	LIB_5976932	PQ457079	Ms Anaregina Araujo, Teresina, Brazil
DRB1*15:129N	LIB_6039214	PQ457081	Ms Anaregina Araujo, Teresina, Brazil
DRB1*16:75	118439	PQ280321	Dr Francisco de Paula Sanchez Gordo, Malaga, Spain
DRB4*01:155	JH0153	PQ621114	Dr Maria Bettinotti, Baltimore, MD, USA
DRB6*02:02	202302799	PQ373828	Dr Amalia Tejeda Velarde, Salamanca, Spain
DQA1*01:04:10	42224118	OR130522	Dr Romain Ferru‐Clement, Poitiers, France
DQA1*01:157	M8267	PQ336900	Dr Thibault Pajot, Lille, France
DQA1*01:160	HG00052681	PQ310452	Histogenetics, Ossining, NY, USA
DQA1*03:01:01:06	24404839	PQ634797	Dr Luis A. Marin, Albacete, Spain
DQA1*03:71	NGR0111	PQ276760	Dr Laura Bungener, Groningen, The Netherlands
DQA1*03:76	022064273	PQ295902	Dr Margot Lepage, Decines‐Charpieu, France
DQA1*04:01:08	502959	PQ463964	Mr Alessandro Pirri, Curitiba, Brazil
DQA1*05:90	donor724700301026	PQ065987	Dr Ahmed Gaballa, Stockholm, Sweden
DQA1*05:116Q	JH0140	PQ196101	Dr Maria Bettinotti, Baltimore, MD, USA
DQB1*05:01:01:03	201‐00289	PQ241144	Ms Yuanli Zhao, Xi'an, China
DQB1*05:01:24:01	201‐00457	PQ476171	Ms Yuanli Zhao, Xi'an, China
DQB1*05:03:01:04	201_01225	PQ241146	Dr Li Zihang, Xi'an, China
DQB1*05:107	46541	PQ196787	Dr Francisco de Paula Sanchez Gordo, Malaga, Spain
DQB1*05:241	LIB_6191294	PQ425362	Ms Anaregina Araujo, Teresina, Brazil
DQB1*06:02:01:16	201‐00326	PQ241147	Ms Yuanli Zhao, Xi'an, China
DQB1*06:02:16	KSR193108	PQ304145	Dr Maria Loginova, Kirov, Russia
DQB1*06:03:01:02	204_00608	PQ241151	Ms Yuanli Zhao, Xi'an, China
DQB1*06:04:01:01	204_00352	PQ241150	Dr Li Zihang, Xi'an, China
DQB1*06:18:01	24819636	PQ451429	Dr Luis A. Marin, Albacete, Spain
DQB1*06:79:01	MC006	PQ276079	Dr Zeying Du, Jacksonville, FL, USA
DQB1*06:490	LIB_6130934	PQ346806	Ms Anaregina Araujo, Teresina, Brazil
DQB1*06:490	LIB_5937018	PQ346812	Ms Anaregina Araujo, Teresina, Brazil
DQB1*02:71	116372	PP826960	Dr Francisco de Paula Sanchez Gordo, Malaga, Spain
DQB1*02:217	RP‐285889	PP977089	Dr Neifi Hassan Saloum Deghaide, Ribeirao Preto, Brazil
DQB1*03:01:01:22	201_01119	PQ476170	Ms Yuanli Zhao, Xi'an, China
DQB1*03:01:01:33	201‐00460	PQ241142	Ms Yuanli Zhao, Xi'an, China
DQB1*03:01:61	LIB_6182338	PQ539776	Ms Anaregina Araujo, Teresina, Brazil
DQB1*03:02:01:25	24819515	PQ383849	Dr Luis A. Marin, Albacete, Spain
DQB1*03:13	204_00651	PQ476167	Ms Yuanli Zhao, Xi'an, China
DQB1*03:127	LIB_5915703	PQ559635	Ms Anaregina Araujo, Teresina, Brazil
DQB1*03:516	PM‐47	PQ497104	Dr Zhangxiang Wanyan, Kunming, China
DQB1*03:547	29001567	PQ497982	Dr Daniel Fuerst, Ulm, Germany
DQB1*04:87	LIB_6167796	PQ539777	Ms Anaregina Araujo, Teresina, Brazil
DPA1*01:196	509483	PQ483444	Mr Alessandro Pirri, Curitiba, Brazil
DPA1*01:224	d13760	PQ438182	Dr Cathi Murphey, San Antonio, TX, USA
DPA1*02:01:01:14	204_00125	PP961284	Ms Yuanli Zhao, Xi'an, China
DPA1*02:02:02:02	PSCDA0202	OR991100	Dr Seik‐Soon Khor, Tokyo, Japan
DPA1*02:02:02:03	PSCDA0202	OR991101	Dr Seik‐Soon Khor, Tokyo, Japan
DPA1*02:03:05	5021391	PQ479082	Prof Renata Zunec, Zagreb, Croatia
DPA1*02:135	HG00055280	PQ619026	Histogenetics, Ossining, NY, USA
DPA1*03:02	d13745	PQ412517	Dr Cathi Murphey, San Antonio, TX, USA
DPA1*04:01:01:03	204‐01440	PQ409245	Ms Yuanli Zhao, Xi'an, China
DPB1*382:01N	LIB_6277733	PQ457080	Ms Anaregina Araujo, Teresina, Brazil
DPB1*582:01	119277	PQ462264	Dr Francisco de Paula Sanchez Gordo, Malaga, Spain
DPB1*1469:01	22‐2081	PQ559524	Assoc Prof Ahmed Mostafa, Saskatoon, Canada
DPB1*1629:01	P15389‐1	PQ535699	Prof Faming Zhu, Hangzhou, China
DPB1*1666:01	81350209	PQ283569	Dr Daniel Fuerst, Ulm, Germany
DPB1*1735:01	24/08208	PQ279895	Mrs Thet Myint, London, UK
DPB1*1747:01	HG00055281	PQ594969	Histogenetics, Ossining, NY, USA
MICA*001:01:01	DUCAF, EJ32B, JS, JVM, L0081785, QBL, VEN	OZ179429, OZ179441, OZ179537, OZ179541, OZ179581, OZ179668, OZ179722	Anthony Nolan Research Institute, London, UK
MICA*002:01:01	591, ELON, GPT, IDF, JBUSH, WDV	OZ179299, OZ179445, OZ179480, OZ179522, OZ179532, OZ179730	Anthony Nolan Research Institute, London, UK
MICA*002:01:02	GU373	OZ179485	Anthony Nolan Research Institute, London, UK
MICA*002:01:03	35841, BON, CAR, ML, GRC‐212, KGU, KT12, KT17, TER‐ND, WT100BIS	OZ179296, OZ179352, OZ179378, OZ179482, OZ179549, OZ179575, OZ179604, OZ179706, OZ179736	Anthony Nolan Research Institute, London, UK
MICA*002:01:07	AP, ARBO, CRB, ELON, WT49	OZ179312, OZ179316, OZ179403, OZ179446, OZ179742	Anthony Nolan Research Institute, London, UK
MICA*002:01:08	DEU, EHM, MARMarg	OZ179419, OZ179438, OZ179623	Anthony Nolan Research Institute, London, UK
MICA*002:01:15	KOSE	OZ179565	Anthony Nolan Research Institute, London, UK
MICA*004:01:01	DOP‐ND	OZ198316	Anthony Nolan Research Institute, London, UK
MICA*004:01:01	BEL5GB, BON, C073, FH05, FH13, MOU, PF97387, PITOUT, TER‐ND, ZM	OZ179326, OZ179353, OZ179368, OZ179451, OZ179461, OZ179635, OZ179659, OZ179661, OZ179707, OZ179752	Anthony Nolan Research Institute, London, UK
MICA*004:01:02	DK1, HOR	OZ179422, OZ179516	Anthony Nolan Research Institute, London, UK
MICA*004:01:03	1563, BJ, DAUDI, RSH, SHJO	OZ179281, OZ179336, OZ179411, OZ179677, OZ179684	Anthony Nolan Research Institute, London, UK
MICA*004:01:06	BM21	OZ179344	Anthony Nolan Research Institute, London, UK
MICA*004:01:09	1416‐1188, AM, BM15, ZM	OZ179278, OZ179304, OZ179341, OZ179753	Anthony Nolan Research Institute, London, UK
MICA*004:01:11	FH11	OZ179457	Anthony Nolan Research Institute, London, UK
MICA*006	KAS116	OZ179545	Anthony Nolan Research Institute, London, UK
MICA*007:01:01	BTB, FH05, HOM‐2, JESTHOM	OZ179360, OZ179452, OZ179514, OZ179534	Anthony Nolan Research Institute, London, UK
MICA*007:01:03	C.M.L.	OZ179396	Anthony Nolan Research Institute, London, UK
MICA*007:01:04	WT24	OZ179738	Anthony Nolan Research Institute, London, UK
MICA*007:01:08	FH06	OZ179454	Anthony Nolan Research Institute, London, UK
MICA*008:01:01	CGM1, C.M.L., COX, FH15, KLO, KNE, LCL721, PF04015, STEINLIN, VAVY, VOO	OZ179392, OZ179397, OZ179400, OZ179465, OZ179558, OZ179562, OZ179589, OZ179657, OZ179695, OZ179720, OZ179726	Anthony Nolan Research Institute, London, UK
MICA*008:01:02	AWELLS, BEL5GB, EK, FMB, HERLUF, SPO010, SSTO, WT47	OZ179322, OZ179327, OZ179443, OZ179469, OZ179500, OZ179691, OZ179693, OZ179740	Anthony Nolan Research Institute, London, UK
MICA*008:01:03	BER, BH, LBF, TL, XLI‐ND	OZ179330, OZ179334, OZ179587, OZ179711, OZ179748	Anthony Nolan Research Institute, London, UK
MICA*008:01:12	PLH	OZ179663	Anthony Nolan Research Institute, London, UK
MICA*008:01:14	BRIP, JAP‐NF	OZ179355, OZ179530	Anthony Nolan Research Institute, London, UK
MICA*008:02	FH06, GEE018, GUA‐ND	OZ179455, OZ179478, OZ179489	Anthony Nolan Research Institute, London, UK
MICA*008:04:01	BM14, CB, EA, FH11, HHKB, JS, KPE, LD2B, WT8, SCHU, SA, MGAR, SAVC	OZ179339, OZ179383, OZ179433, OZ179458, OZ179507, OZ179538, OZ179567, OZ179593, OZ179625, OZ179678, OZ179680, OZ179682, OZ179746	Anthony Nolan Research Institute, London, UK
MICA*008:04:02	AM, APD, DKB, EMJ, HID, MADURA, MT14B, PE117, SLE005, SNA‐BLL	OZ179305, OZ179310, OZ179425, OZ179449, OZ179509, OZ179619, OZ179637, OZ179650, OZ179687, OZ179689	Anthony Nolan Research Institute, London, UK
MICA*008:04:05	CC	OZ179386	Anthony Nolan Research Institute, London, UK
MICA*008:13:01	FH13, RAJI	OZ179462, OZ179670	Anthony Nolan Research Institute, London, UK
MICA*009:01:01	BOB, BRIP, C047, C073, LATIF, TUBO	OZ179348, OZ179356, OZ179364, OZ179369, OZ179585, OZ179715	Anthony Nolan Research Institute, London, UK
MICA*009:01:03	JHAF	OZ179536	Anthony Nolan Research Institute, London, UK
MICA*009:01:04	AKIBA, BGE, C1R, E4181324, EHM, HARA, HERLUF, KAWASAKI, KHAGNI, KIME, KT12, LK707, TOKUNAGA	OZ179301, OZ179332, OZ179372, OZ179431, OZ179439, OZ179493, OZ179501, OZ179547, OZ179551, OZ179554, OZ179576, OZ179599, OZ179718	Anthony Nolan Research Institute, London, UK
MICA*009:01:05	LCL721	OZ179590	Anthony Nolan Research Institute, London, UK
MICA*009:02:01	KLO, MANIKA, SHJO	OZ179559, OZ179621, OZ179685	Anthony Nolan Research Institute, London, UK
MICA*010:01:01	1416‐1188, C047, FH15, KAS011	OZ179279, OZ179365, OZ179466, OZ179543	Anthony Nolan Research Institute, London, UK
MICA*010:01:02	BSM, DES.DI, MJ4, MLF	OZ179358, OZ179417, OZ179627, OZ179629	Anthony Nolan Research Institute, London, UK
MICA*010:01:03	BOLETH	OZ179350	Anthony Nolan Research Institute, London, UK
MICA*010:01:04	CB6B	OZ179381	Anthony Nolan Research Institute, London, UK
MICA*010:01:05	AP, ISH3, T7526, T7527, TAB089	OZ179313, OZ179526, OZ179699, OZ179701, OZ179703	Anthony Nolan Research Institute, London, UK
MICA*010:01:09	GEE018, KRC‐110	OZ179479, OZ179573	Anthony Nolan Research Institute, London, UK
MICA*011:01:01	CGM1, EC, HO301, IBW9, LWAGS, MZ070782, PA, PMG075	OZ179393, OZ179434, OZ179512, OZ179520, OZ179612, OZ179639, OZ179645, OZ179665	Anthony Nolan Research Institute, London, UK
MICA*011:01:04Q	PMG075	OZ179666	Anthony Nolan Research Institute, London, UK
MICA*012:01:01	VEN	OZ179723	Anthony Nolan Research Institute, London, UK
MICA*012:01:02	APA, AT, BUR,E, HAS‐15, KY, VOO	OZ179307, OZ179319, OZ179362, OZ179497, OZ179579, OZ179727	Anthony Nolan Research Institute, London, UK
MICA*016:01:01	BJ, FPAF, J0528239, KPE, OZB, TISI, TL	OZ179337, OZ179472, OZ179528, OZ179568, OZ179642, OZ179709, OZ179712	Anthony Nolan Research Institute, London, UK
MICA*017:01	BEI, CEK008AN, DBB, DEM, FMB, G088, MOLT‐4, WIN, WJR076	OZ179324, OZ179387, OZ179413, OZ179415, OZ179470, OZ179474, OZ179631, OZ179732, OZ179734	Anthony Nolan Research Institute, London, UK
MICA*018:01:01	CTM1953541, DO208915, IDF, MOLT‐4	OZ179406, OZ179427, OZ179523, OZ179632	Anthony Nolan Research Institute, London, UK
MICA*018:01:03	BM16	OZ179342	Anthony Nolan Research Institute, London, UK
MICA*018:01:10	35020	OZ179291	Anthony Nolan Research Institute, London, UK
MICA*019:01:01	CF996, WT51	OZ179390, OZ179743	Anthony Nolan Research Institute, London, UK
MICA*019:01:02	APA	OZ179308	Anthony Nolan Research Institute, London, UK
MICA*027:01:01	SWEIG007	OZ179697	Anthony Nolan Research Institute, London, UK
MICA*027:01:02	32511, CALOGERO, DAN723, EC, G088, GRC‐212, HGOM, HID, KIME, LKT14, PE117, XLI‐ND	OZ179288, OZ179374, OZ179408, OZ179435, OZ179475, OZ179483, OZ179505, OZ179510, OZ179555, OZ179603, OZ179651, OZ179749	Anthony Nolan Research Institute, London, UK
MICA*029:01:01	LK707	OZ179600	Anthony Nolan Research Institute, London, UK
MICA*041	M7	OZ179615	Anthony Nolan Research Institute, London, UK
MICA*046	M7	OZ179616	Anthony Nolan Research Institute, London, UK
MICA*049:01:01	AT, LKT2, LUY	OZ179320, OZ179607, OZ179610	Anthony Nolan Research Institute, London, UK
MICA*110	35020	OZ179292	Anthony Nolan Research Institute, London, UK
MICA*250:01:01	PETCH	OZ179654	Anthony Nolan Research Institute, London, UK
MICB*002:01:01	BOLETH	OZ179351	Anthony Nolan Research Institute, London, UK
MICB*002:01:02	1416‐1188, AMALA, BSM, BUR,E, CAR, ML, DES.DI, DKB, FH15, G088, KNE, LZL, MADURA, MJ4, MLF, TER‐ND, WT100BIS	OZ198369, OZ198370, OZ198372, OZ198373, OZ198374, OZ198377, OZ198378, OZ198379, OZ198380, OZ198382, OZ198383, OZ198384, OZ198385, OZ198386, OZ198389, OZ198390	Anthony Nolan Research Institute, London, UK
MICB*002:01:11	BOB	OZ179349	Anthony Nolan Research Institute, London, UK
MICB*002:01:18	DO208915, IDF, MOLT‐4	OZ179428, OZ179525, OZ179633	Anthony Nolan Research Institute, London, UK
MICB*002:01:27	TISI, TL	OZ179710, OZ179713	Anthony Nolan Research Institute, London, UK
MICB*002:01:28	DOP‐ND	OZ198317	Anthony Nolan Research Institute, London, UK
MICB*002:01:28	32367, GUA‐ND	OZ179285, OZ179491	Anthony Nolan Research Institute, London, UK
MICB*004:01:01	AM, BM14, CB, DAN723, DK1, FH11, HARA, HHKB, JS, LD2B, MGAR, MT14B, PE117, SAVC, SA, SCHU, VEN, WT8	OZ179306, OZ179340, OZ179384, OZ179409, OZ179423, OZ179460, OZ179494, OZ179508, OZ179539, OZ179594, OZ179626, OZ179638, OZ179652, OZ179679, OZ179681, OZ179683, OZ179724, OZ179747	Anthony Nolan Research Institute, London, UK
MICB*004:01:01	CAM020, PETCH	OZ179376, OZ179655	Anthony Nolan Research Institute, London, UK
MICB*004:01:02	APD	OZ179311	Anthony Nolan Research Institute, London, UK
MICB*004:01:06	DHIF, EHM, MARMarg	OZ179421, OZ179440, OZ179624	Anthony Nolan Research Institute, London, UK
MICB*004:01:11	M7, PLH	OZ179617, OZ179664	Anthony Nolan Research Institute, London, UK
MICB*004:01:12	GU373, RAJI	OZ198381, OZ198388	Anthony Nolan Research Institute, London, UK
MICB*004:01:13	KPE	OZ179569	Anthony Nolan Research Institute, London, UK
MICB*005:01:02	J0528239, KPE, OZB	OZ179529, OZ179570, OZ179644	Anthony Nolan Research Institute, London, UK
MICB*005:02:01	DUCAF, JS, JVM, L0081785, QBL, VEN	OZ179430, OZ179540, OZ179542, OZ179582, OZ179669, OZ179725	Anthony Nolan Research Institute, London, UK
MICB*005:02:03	SSTO	OZ179694	Anthony Nolan Research Institute, London, UK
MICB*005:02:05	35841, BM21, CRB, DK1, ELON, GU373, HOR, ZM	OZ179297, OZ179345, OZ179405, OZ179424, OZ179447, OZ179488, OZ179517, OZ179754	Anthony Nolan Research Institute, London, UK
MICB*005:02:06	AWELLS, BEL5GB, BM9, BTB, C047, C1R, EK, ELON, HERLUF, HOM‐2, JESTHOM, KAS116, LCL721, PF97387, WT24	OZ179323, OZ179328, OZ179347, OZ179361, OZ179366, OZ179373, OZ179444, OZ179448, OZ179502, OZ179515, OZ179535, OZ179546, OZ179591, OZ179660, OZ179739	Anthony Nolan Research Institute, London, UK
MICB*005:02:07	C047, CALOGERO	OZ179367, OZ179375	Anthony Nolan Research Institute, London, UK
MICB*005:02:13	BEL5GB, BON, FH05, LAM, MOU, PITOUT	OZ179329, OZ179354, OZ179453, OZ179583, OZ179636, OZ179662	Anthony Nolan Research Institute, London, UK
MICB*005:02:21	26/27, AP, CB6B, DAN723, EC, GRC‐212, HAS‐15, HGOM, HID, ISH3, KRC‐005, KRC‐110, KT12, KT17, T7526, T7527, TAB089	OZ179283, OZ179314, OZ179382, OZ179410, OZ179436, OZ179484, OZ179498, OZ179506, OZ179511, OZ179527, OZ179571, OZ179574, OZ179577, OZ179605, OZ179700, OZ179702, OZ179704	Anthony Nolan Research Institute, London, UK
MICB*005:02:27	EC, HO301, LWAGS, MZ070782, PMG075	OZ179437, OZ179513, OZ179613, OZ179640, OZ179667	Anthony Nolan Research Institute, London, UK
MICB*005:02:28	C.M.L., DEU	OZ179398, OZ179420	Anthony Nolan Research Institute, London, UK
MICB*005:02:32	APA, BM92, CAR, ML, HAS‐15, KGU, KHAGNI, KIME, KOSE, KT12, LATIF, KT17, LKT3, RML, VOO, WT52	OZ179309, OZ179346, OZ179380, OZ179499, OZ179550, OZ179552, OZ179556, OZ179566, OZ179578, OZ179586, OZ179606, OZ179609, OZ179672, OZ179728, OZ179745	Anthony Nolan Research Institute, London, UK
MICB*005:02:39	HERLUF	OZ179503	Anthony Nolan Research Institute, London, UK
MICB*005:03:01	KAWASAKI	OZ179548	Anthony Nolan Research Institute, London, UK
MICB*005:03:04	AT, LKT2	OZ179321, OZ179608	Anthony Nolan Research Institute, London, UK
MICB*005:06:01	KLO, MANIKA, SHJO	OZ179560, OZ179622, OZ179686	Anthony Nolan Research Institute, London, UK
MICB*008:01:01	CGM1, C.M.L., COX, CTM1953541, FH13, FH15, KLO, KNE, PF04015, STEINLIN, VAVY, VOO	OZ179395, OZ179399, OZ179401, OZ179407, OZ179464, OZ179468, OZ179561, OZ179564, OZ179658, OZ179696, OZ179721, OZ179729	Anthony Nolan Research Institute, London, UK
MICB*008:01:11	AP	OZ179315	Anthony Nolan Research Institute, London, UK
MICB*008:01:14	DOP‐ND	OZ198318	Anthony Nolan Research Institute, London, UK
MICB*008:01:18	GUA‐ND	OZ179492	Anthony Nolan Research Institute, London, UK
MICB*009:01:04N	26/27	OZ179284	Anthony Nolan Research Institute, London, UK
MICB*009:01:09N	CAM020, HS67, PAR	OZ179377, OZ179519, OZ179648	Anthony Nolan Research Institute, London, UK
MICB*014:01:01	EMJ, PETCH, SLE005, SNA‐BLL	OZ179450, OZ179656, OZ179688, OZ179690	Anthony Nolan Research Institute, London, UK
MICB*014:01:09	LIE, PAR, RSA‐ND	OZ179598, OZ179649, OZ179676	Anthony Nolan Research Institute, London, UK

**TABLE 3:** Recently published sequences.

HLA allele	References
A*01:01:23	(Morozova, Loginova, and Paramonov 2024)
A*01:467	(Loginova, Obukhov, and Paramonov 2024a)
A*02:01:222	(Ananeva, Akhmetshina, et al. 2024)
A*02:06:12	(Morozova, Loginova, and Paramonov 2024)
A*02:110	(Morozova, Loginova, and Paramonov 2024)
A*02:406	(J. P. Li, Wei, and Li 2024)
A*02:407	(X. F. Li, Zhang, Lin, Wei, and Li 2024)
A*02:629	(Morozova, Loginova, and Paramonov 2024)
A*02:889	(Xia and Li 2024)
A*02:1138	(Ren et al. 2024)
A*02:1188	(Loginova, Obukhov, and Paramonov 2024a)
A*02:1189	(Blandin, Wojciechowski, et al. 2024)
A*03:01:01:121	(Moya‐Quiles and Muro 2024a)
A*03:01:01:122	(Y. Han, Li, et al. 2024)
A*03:452	(Tran et al. 2023)
A*11:01:01:07	(Y. Chen et al. 2024)
A*11:72	(Morozova, Loginova, and Paramonov 2024)
A*11:83	(Morozova, Loginova, and Paramonov 2024)
A*11:454	(Morozova, Loginova, and Paramonov 2024)
A*11:479N	(K. L. Yang and Lin 2024b)
A*23:147	(Giorno‐Amar et al. 2024)
A*24:02:01:144Q	(Stelet, Silva, et al. 2024)
A*24:02:15	(Morozova, Loginova, and Paramonov 2024)
A*24:02:51	(Morozova, Loginova, and Paramonov 2024)
A*24:02:169	(Elsermans, Cargou, et al. 2024)
A*24:146	(Morozova, Loginova, and Paramonov 2024)
A*25:90	(Loginova, Obukhov, and Paramonov 2024a)
A*25:91	(Loginova, Obukhov, and Paramonov 2024a)
A*26:01:81	(D. H. Han et al. 2024)
A*29:02:01:41	(Moya‐Quiles and Muro 2024a)
A*29:77	(Morozova, Loginova, and Paramonov 2024)
A*30:02:01:24	(Moya‐Quiles and Muro 2024a)
A*30:95	(Morozova, Loginova, and Paramonov 2024)
A*31:217	(Baek et al. 2024a)
A*32:01:60	(Loginova, Obukhov, and Paramonov 2024a)
A*33:03:68	(F. Yang et al. 2024)
A*36:15	(de Oliveira et al. 2024)
B*07:02:104	(Ananeva, Akhmetshina, et al. 2024)
B*07:81	(Morozova, Loginova, and Paramonov 2024)
B*07:163	(Morozova, Loginova, and Paramonov 2024)
B*07:516:01:01	(Loginova, Smirnova, and Paramonov 2024)
B*08:01:01:75	(Minshall et al. 2024)
B*13:01:01:12	(Agarwal et al. 2024)
B*13:53	(Morozova, Loginova, and Paramonov 2024)
B*15:358	(J. P. Li, Zhang, et al. 2024)
B*15:359	(X. F. Li, Zhang, Lin, and Li 2024)
B*15:675	(Tran et al. 2023)
B*15:693	(Davies et al. 2024)
B*27:264	(Baek et al. 2024b)
B*35:01:01:89	(Minshall et al. 2024)
B*35:20:03	(Molinari et al. 2024)
B*37:107	(Baek et al. 2024c)
B*37:113	(Loginova, Obukhov, and Paramonov 2024b)
B*39:79	(Morozova, Loginova, and Paramonov 2024)
B*39:130	(L. Li and Tian 2024a)
B*39:223	(Loginova, Smirnova, and Paramonov 2024)
B*40:01:90	(Loginova, Smirnova, and Paramonov 2024)
B*40:02:05	(Morozova, Loginova, and Paramonov 2024)
B*40:78	(K. L. Yang and Lin 2024a)
B*40:96	(Zhou et al. 2024)
B*40:125:01	(Morozova, Loginova, and Paramonov 2024)
B*40:186:02	(Tian and Li 2024a)
B*40:298:02	(Tian and Li 2024b)
B*40:523	(Baek et al. 2024d)
B*40:585	(N. Liu, Wang, Jia, and Sun 2024)
B*40:587	(Blandin, Visentin, et al. 2024)
B*41:02:12	(Loginova, Obukhov, and Paramonov 2024b)
B*44:02:01:87	(Minshall et al. 2024)
B*44:03:39	(Morozova, Loginova, and Paramonov 2024)
B*44:418	(Cheranev et al. 2024)
B*46:01:25	(L. Li and Tian 2024b)
B*48:01:12	(Tran et al. 2023)
B*48:01:14	(Y. Han, Jiao, and Yang 2024)
B*48:55	(Tran et al. 2023)
B*51:01:01:123	(Minshall et al. 2024)
B*51:377N	(Baek et al. 2024e)
B*51:409N	(Lion et al. 2024)
B*53:80N	(Loginova, Smirnova, and Paramonov 2024)
B*55:39	(K. L. Yang and Lin 2024d)
B*57:01:01:31	(Minshall et al. 2024)
B*57:01:02	(Morozova, Loginova, and Paramonov 2024)
B*57:90	(Morozova, Loginova, and Paramonov 2024)
B*58:139	(Q. Li et al. 2024)
C*01:262	(Pajot et al. 2024)
C*02:56	(Morozova, Loginova, and Paramonov 2024)
C*03:139:01	(Morozova, Loginova, and Paramonov 2024)
C*03:656	(Shen et al. 2024)
C*03:694	(Loginova, Morozova, and Paramonov 2024)
C*04:01:01:182	(Kouniaki, Athanassiades, and Tsirogianni 2024)
C*04:105N	(Morozova, Loginova, and Paramonov 2024)
C*04:524	(Loginova and Paramonov 2024)
C*04:535	(Okumura et al. 2024)
C*05:18:01	(Morozova, Loginova, and Paramonov 2024)
C*05:277	(Tran et al. 2023)
C*05:298	(Loginova, Dudina, and Paramonov 2024)
C*05:301	(Loginova, Morozova, and Paramonov 2024)
C*06:391	(AlAbduladheem et al. 2024)
C*07:01:126	(Elsermans, Visentin, et al. 2024)
C*07:02:66	(Morozova, Loginova, and Paramonov 2024)
C*07:02:156	(Ananeva, Akhmetshina, et al. 2024)
C*07:247	(Morozova, Loginova, and Paramonov 2024)
C*07:971	(Gatouillat et al. 2024)
C*07:1041	(Tran et al. 2023)
C*07:1043	(Tran et al. 2023)
C*07:1153	(Ananeva, Sergeeva, and Shagimardanova 2024)
C*07:1154	(Ananeva, Sergeeva, and Shagimardanova 2024)
C*07:1160Q	(Loginova, Morozova, and Paramonov 2024)
C*12:419	(Huang et al. 2024)
C*12:424	(Glover et al. 2024)
C*14:152	(Kim, Yang, and Oh 2024)
C*14:153	(J. J. Yang, Kim, and Oh 2024)
E*01:03:01:16	(Minshall et al. 2024)
E*01:03:02:41	(Minshall et al. 2024)
E*01:151	(Dong et al. 2024)
G*01:01:03:06	(Minshall et al. 2024)
G*01:06:01:02	(Mujumdar, Thakur, Bhor, et al. 2024)
G*01:24:02	(Mujumdar, Thakur, Talge, et al. 2024)
DRB1*01:01:30	(Morozova, Loginova, and Paramonov 2024)
DRB1*01:151	(Loginova, Dudina, and Paramonov 2024)
DRB1*03:218N	(Athanassiades et al. 2024)
DRB1*04:04:21	(Loginova, Makhova, and Paramonov 2024)
DRB1*04:21	(Morozova, Loginova, and Paramonov 2024)
DRB1*04:388	(N. Liu, Wang, Sun, and Jia 2024)
DRB1*08:131	(Loginova, Makhova, and Paramonov 2024)
DRB1*11:10:02	(Morozova, Loginova, and Paramonov 2024)
DRB1*11:42:01	(Morozova, Loginova, and Paramonov 2024)
DRB1*11:137	(Morozova, Loginova, and Paramonov 2024)
DRB1*12:01:13	(Loginova, Makhova, and Paramonov 2024)
DRB1*14:27:01	(Morozova, Loginova, and Paramonov 2024)
DRB1*14:249	(Tran et al. 2023)
DRB1*14:270	(Leonov et al. 2024)
DRB1*15:233	(Loginova, Makhova, and Paramonov 2024)
DRB1*16:02:13	(K. L. Yang and Lin 2024c)
DRB1*16:79	(Andrade et al. 2024)
DRB3*02:185	(Zhao, Webster, Gorkoff, et al. 2024)
DRB4*01:03:01:21	(Z. Li et al. 2024)
DRB5*01:130	(Tran et al. 2023)
DRB5*02:02:05	(Blandin, Ralazamahaleo, et al. 2024)
DRB5*02:37	(Tran et al. 2023)
DQA1*01:02:15	(Tran et al. 2023)
DQA1*01:02:16	(Tran et al. 2023)
DQA1*01:04:08	(Tran et al. 2023)
DQA1*01:173	(Moya‐Quiles and Muro 2024b)
DQA1*02:01:15Q	(Tran et al. 2023)
DQA1*03:82	(Moya‐Quiles and Muro 2024b)
DQA1*04:01:07	(Tran et al. 2023)
DQA1*05:01:16	(J. Liu et al. 2024)
DQA1*05:05:16:01	(Tran et al. 2023)
DQA1*05:05:16:02	(Tran et al. 2023)
DQA1*05:05:23	(Ocejo‐Vinyals et al. 2024)
DQA1*05:73	(Gil‐Etayo et al. 2024)
DQA1*06:01:04	(Gaballa, Soderstrom, Lundin, and Eich 2024)
DQB1*05:02:01:19	(Kouniaki, Tarassi, and Tsirogianni 2024)
DQB1*05:42	(Morozova, Loginova, and Paramonov 2024)
DQB1*06:01:08	(Morozova, Loginova, and Paramonov 2024)
DQB1*06:03:51	(Ananeva, Akhmetshina, et al. 2024)
DQB1*06:432	(J. J. Yang and Oh 2024)
DQB1*02:02:01:14	(Zhao, Webster, Pelzer, et al. 2024)
DQB1*03:01:01:69	(Kouniaki and Tsirogianni 2024)
DQB1*03:524	(Loginova and Paramonov 2024)
DQB1*03:562	(Ocejo‐Vinyals et al. 2024)
DQB1*04:01:01:03	(Y. Li et al. 2024)
DPA1*01:03:01:81	(Marin Rubio and Ontanon 2024)
DPA1*01:03:01:89	(Marin Rubio and Ontanon 2024)
DPA1*01:03:01:90	(Marin Rubio and Ontanon 2024)
DPA1*01:03:01:91	(Marin Rubio and Ontanon 2024)
DPA1*01:03:01:93	(Marin Rubio and Ontanon 2024)
DPA1*01:03:01:94	(Marin Rubio and Ontanon 2024)
DPA1*01:03:38:02	(Tran et al. 2023)
DPA1*01:03:45	(Tran et al. 2023)
DPA1*01:136	(Tran et al. 2023)
DPA1*01:137N	(Tran et al. 2023)
DPA1*01:204	(He et al. 2024)
DPA1*01:214	(de Lima et al. 2024)
DPA1*01:222	(Leonov et al. 2024)
DPA1*02:01:01:39	(Gao et al. 2024)
DPA1*02:02:13	(Tran et al. 2023)
DPA1*02:96	(Tran et al. 2023)
DPA1*02:141	(Stelet, Lemos, et al. 2024)
DPA1*02:143	(Lee et al. 2024)
DPA1*02:146	(B. Chen et al. 2024)
DPB1*01:01:15	(Oh et al. 2024)
DPB1*03:01:27	(Muniyappa et al. 2024)
DPB1*05:01:22	(Xie et al. 2024)
DPB1*1327:01	(Zhao, Webster, Pearce, et al. 2024)
DPB1*1641:01	(Bian et al. 2024)

The DRB1*15:13 allele has had a Q suffix added in December 2024, now making it DRB1*15:13Q, as it was recognised in having the same amino acid deletion as had previously been described in the DRB1*11:272Q allele. The C*04:09N allele has had its suffix changed from N to L in December 2024 and is now designated C*04:09L based on information published recently indicating that this allele showed detectable levels of expression (Welters et al. 2024). A further 6 alleles, MICA*023, MICA*026, MICA*036, MICB*005:04, MICB*005:02:02 and MICB*002:01:34 were all deleted in October 2024 following the resequencing of the original material in which these alleles were first described.

All new and confirmatory sequences should now be submitted directly to the WHO Nomenclature Committee for Factors of the HLA System via the IPD‐IMGT/HLA Database using the sequence submission tool provided (Barker et al. 2023; Robinson, Barker, and Marsh 2024). The IPD‐IMGT/HLA Database may be accessed via the World Wide Web at: www.ebi.ac.uk/ipd/imgt/hla

## Conflicts of Interest

The author declares no conflicts of interest.

## Data Availability

The authors have nothing to report.
